# Middle aged and older adult’s perspectives of their own home environment: a review of qualitative studies and meta-synthesis

**DOI:** 10.1186/s12877-023-04279-1

**Published:** 2023-10-31

**Authors:** Roslyn Aclan, Stacey George, Heather Block, Rachel Lane, Kate Laver

**Affiliations:** 1 College of Medicine and Public Health, Flinders University, Adelaide, Australia; 2 Occupational Therapy, Academic Lead and Research Lead, Allied Health Chair, Northern Adelaide Local Health Network, College of Nursing and Health Sciences, Flinders University, Adelaide, Australia; 3 College of Nursing and Health Sciences, Flinders University, Adelaide, Australia; 4 Department of Rehabilitation, Aged and Extended Care, College of Medicine and Public Health, Flinders University, Adelaide, Australia

**Keywords:** Age-in-place, Housing, Views, Meta-synthesis

## Abstract

**Background:**

Most people prefer to remain in their homes and communities as long as possible. Staying at home is widely beneficial as ageing within the home promotes independence and costs less than residential aged care. Understanding meanings and drivers of remaining at home is an area of importance.

**Objective:**

The objective of this systematic review of qualitative studies was to synthesise middle and older aged adult’s perspective of their home environment and determine the factors that are important when making decisions about future housing.

**Methods:**

This review and meta-synthesis was conducted in accordance with JBI (formally known as the Joanna Briggs Institute) methodology for systematic reviews of qualitative evidence. Meta-aggregation was used as the method of synthesis. Included qualitative studies involved middle and older aged adults and their views about ageing and housing. Published studies were identified in four electronic databases and grey literature. Critical appraisal and extraction were conducted using JBI tools and findings were categorised and synthesised into findings.

**Results:**

A total of 46 papers with 5183 participants on the concept of home were included. Most of the participants were older (> 65 years old) and the perspectives of middle-aged people were largely absent. Factors impacting on future housing decisions among individuals were identified. Seven synthesized findings emerged—independence, finances, stigma, attitudes towards ageing, attachments with home, aesthetics, and family connection.

**Conclusion:**

Older people have a greater sense of independence and autonomy if they remain in their own home. Multiple external factors impacted on their perspectives including a sense of stigma about ageing, fear of being a burden to others and their own financial position which in some cases restricted their options. This review provides a comprehensive description of the different factors that need to be considered when planning future housing needs; both for individuals and for communities.

**Supplementary Information:**

The online version contains supplementary material available at 10.1186/s12877-023-04279-1.

## Background

Globally, there are an estimated 1 billion older people aged 60 years and older [1]. This number is expected to rise and nearly double from 12% of the population to 22%, due to continued decline in fertility rates and increased life expectancy [2]. Functional ability is determined based on the intrinsic capacity of the individual, the environment a person lives in and how they interact within their environment [3]. Ageing can reduce a person’s intrinsic capacity thereby reducing their functional ability [4]. As a result, their ability to live independently may be compromised. When this occurs, support may be needed to help the person ‘age in place’ through modification of the home (e.g., with ramps or rails).

A review by Pani-Harreman et al. [5] found  that ‘Ageing in place’ refers not only to the characteristics of the home but also to social and support networks that surround the person. Most people prefer to remain in their homes for as long as possible and supporting ageing in place (within the home) is much less expensive for governments than funding residential care places [6–10]. Research suggests that formal support or home is an effective method of enabling the ageing population to remain at home [11, 12]. Many countries are now attempting to improve the provision of home care to support ageing in place, rather than investing in residential care facilities [13]. Worldwide, the expenditure on long term care is expected to increase from 1.5% in 2010 to more than 3% in 2050 [11]. For example, in Australia, it costs $85,818 total operational costs per residential aged care bed per annum, versus $26,382per annum for the cost of home care packages [14, 15]. Other research shows, in Germany, the average cost of nursing home care is $49,219 per annum, whilst long term care costs an average of $43,997 per annum [16]. Similarly, in America, the average cost of home care per month is approximately $3500 per month versus $7000 per month for the cost of care in a nursing home [17]. Previous studies show that older adults have a strong emotional attachment to their home and the home is not just a building but a place of meaning [18, 19]. Qualitative research has described a clear relationship between older people, their physical environment, and their personal views about ageing [20, 21]. Home is usually considered a place of comfort and freedom, independence, and safety [7, 10, 20, 21]. Living at home provides older adults with a sense of being anchored to their living environment and a sense of individuality where they are able to decorate/alter their home or fulfil valued roles and activities [10]. Possessions within the home provoke memories and create opportunities for self-reflection [21]. Being close to family, friends, neighbours, social activities, and local shops contributes towards a positive ageing process [8–10]. In contrast, Aplin, Canagasuriam [20], reported that for younger adults (aged 39–40 years old), home is a place for functionality and comfort.

Most of the research in this field has been conducted with older people who may already be experiencing disability and loss of function due to ageing. Less is known about the views that older adults have about ageing at home prior to the onset of illness or disability. This is important in order to inform healthy ageing interventions and help older people to maintain independence and participation in the community. Furthermore, there is a dearth of research regarding the perspectives of middle-aged people and their longer-term plans for housing. Adults are unlikely to be considering home modifications (such as ramps or rails) in middle age however, they may be considering longer term needs when planning renovations or considering downsizing once children leave home. Understanding the value of and meanings of home in both middle aged and older adults is an area of critical importance, and the synthesis of existing literature has not yet been done. This review seeks to explore what home means to middle and older aged adults. The aim of this qualitative meta-synthesis systematic review is to synthesize and understand middle and older adult’s perspective of their home environment and concept of home to determine the factors that are important when making decisions about their future housing.

## Methods

This review followed the JBI methodology for systematics review of qualitative evidence [22]. The protocol for this review was developed 'a priori' and stored in an institutional repository, see ‘availability of data and materials’ [23]. The preferred reporting items for Systematic reviews and Meta-Analyses (PRISMA) statement adhered, see supplementary material, Additional file 1 [24].

### Inclusion criteria

Articles were included if a) they included middle (aged over 50) and older aged adults (aged over 65) (either within the metropolitan or rural area) in any country, b) explored personal experiences, beliefs, and attitudes towards ageing within the home, c) used qualitative methodologies, d) in any community setting, e) were published from 2005 to 2022 to represent contemporary literature. Studies which focused on specific diagnostic populations (e.g., post hip fracture) were excluded. Studies were excluded if they were not in English. Mixed method studies were only considered if data from the qualitative components could be extracted.

### Search strategy and study selection

The search aimed to find both published and unpublished studies. Full search strategies are detailed in the supplementary material, Additional file 2. The reference lists of all eligible studies were screened for additional studies. Initial database searches occurred on 19 May 2021 and 12 July 2022. The search strategy was verified by an experienced academic librarian and translated each database. Databases searched were Medline, PyscInfo (Ovid), Scopus (Elsevier) and CINAHL (EBSCOhost). Sources of unpublished studies and grey literature searched were Google Scholar and Council on the Ageing (COTA), ProQuest Dissertations and Theses and WorldWideScience.org. All identified citations were collated and uploaded into Endnote X9.3 [25] then transferred to Covidence where duplicates were removed.

### Quality appraisal

Studies were assessed by two reviewers independently to rate the methodological quality of the studies using the standardised JBI Critical Appraisal Checklist for Qualitative Research located in JBI SUMARI [26]. All included studies underwent data extraction and synthesis, where possible, in order to employ an inclusive approach with diverse studies and datasets [27].

### Data screening and extraction

Two reviewers (RD and KL) independently screened titles and abstracts and selected those that appeared to meet the inclusion criteria for full text review. The same process involving two reviewers was conducted for review of full text. Any disagreements between the two reviewers were resolved by discussion and/or consultation with a third reviewer to arrive at a consensus. Included studies were imported into the JBI System for Unified Management, Assessment and Review of Information (JBI SUMARI) for extraction and synthesis [26]. Qualitative data were extracted by one author (RD) using the standardised JBI data extraction tool. Data extracted included specific details about the populations, context, culture, geographical location, study methods and the phenomena of interest relevant to the review question and specific objectives (supplementary material, Additional file 3). Findings (a verbatim extract of the author’s interpretation of results) and illustrations (direct participant quotes) were extracted from the included studies into JBI SUMARI.. Findings and illustrations were extracted by the primary reviewer (RD) and confirmed by the secondary reviewer (KL) after thorough review of the papers.

### Meta-synthesis

Extracted findings were categorised based on meaning Findings were aggregated into categories and grouped into synthesised findings using the JBI meta-aggregative approach [26–28]. In a meta-aggregation, the author does not re-interpret the findings of included studies but instead synthesizes and accurately presents the findings as reported by the original authors [29]. Once findings are extracted and allocated a level of credibility, they are grouped (on the basis of having similar meaning or concept) and then combined into synthesized findings (where each synthesized finding contains at least two categories) [28]. The final categories and synthesised findings were discussed by three reviewers (RD, KL and HB) and revised until consensus was reached.

## Results

### Characteristics of included studies

**Fig. 1:**
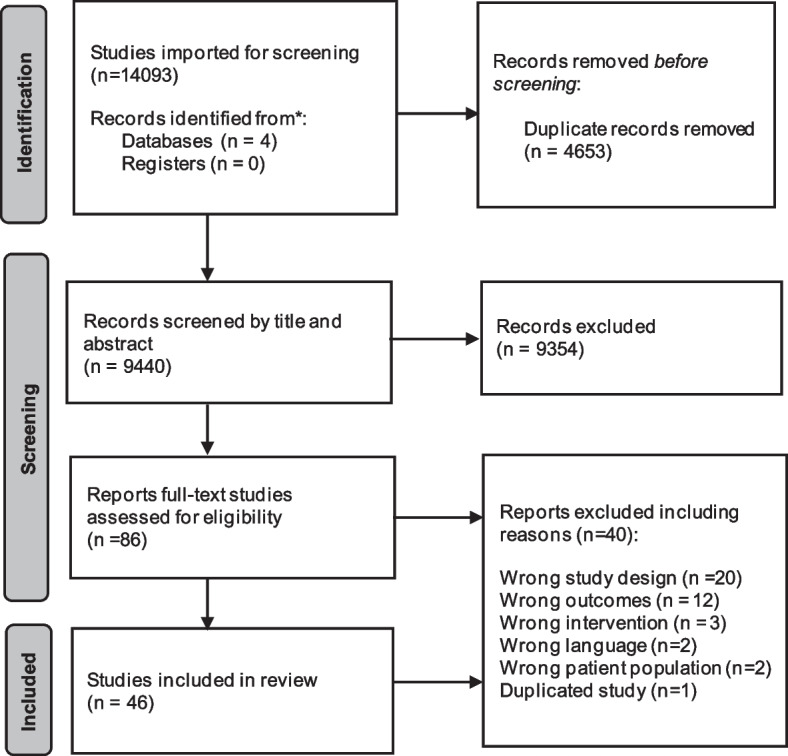
PRISMA flow chart [24]

The search yielded 14,093 studies. In total, 4653 duplicates were removed, 9440 titles and abstracts were screened. Of these, 86 studies were reviewed in full text and 46 studies were included in the review. See PRISMA Fig. 1 [24]. Included studies were published between 2006–2022 in 15 countries: Australia, Canada, United States, Norway, Spain, Sweden, Finland, United Kingdom, Malaysia, Korea, New Zealand, France, India, Brazil, and China. The number of participants in the studies ranged from 10–1680. All studies collected data through focus groups, semi-structured/in-depth interviews, surveys, photo diaries and field notes. Overall, the findings comprised the data for 5183 middle or older aged adults. Twelve studies included participant groups comprising both middle aged and older adults [30–41]. However, middle aged adults were in the minority and their data were not analysed or presented separately. Of the 12 studies that included middle aged participants, these studies included a large age range, varying from 50–92 years old.

### Methodological quality

The methodological quality of the 46 studies is summarised in the supplementary material, Additional file 4 [42]. Four studies met the criteria 100% of the time and the remaining 42 met the criteria 80% of the time. Overall, the methodological quality of the 46 eligible studies was considered good and no studies were excluded following critical appraisal.

### Data extraction and meta-synthesis

A total of 429 findings were extracted and categorised into 17 categories (see Supplementary table 1–7, Additional file 5). The 17 categories were synthesised into seven synthesized findings (see Table 1 for full details). A total of 12 out of 46 studies included perspectives of middle-aged participants over the aged of 50.

**Table 1:** Summary of synthesized findings

**Synthesized finding 1:** People value independence, autonomy and housing that fits with their functional abilities	Category 1.1- Individuals perceived themselves as “independent” if they remained in their own home
	Category 1.2- The need for the “right fit” between an older person’s abilities and the environment
**Synthesized finding 2:** Finances and costs constrained decisions about future housing decisions	Category 2.1- Financial resources were a factor when making housing decisions
**Synthesized finding 3:** People experienced feelings of stigma regarding ageing and were concerned about being a burden	Category 3.1- Negative views associated with moving house
	Category 3.2- Individuals did not want to be a burden
	Category 3.3- Stigma
	Category 3.4- Acceptance of ageing impacted housing decisions
**Synthesized finding 4:** People experienced both positive and negative attitudes to future housing	Category 4.1- Individuals with positive attitudes to future housing
	Category 4.2- Some individuals avoid thinking about future housing
**Synthesized finding 5:** Emotions, meaningful activities and attachments to the home played a part in housing decisions	Category 5.1- Emotions related to home
	Category 5.2- Meaningful activities within the home
	Category 5.3- Strong sense of attachment to the home
**Synthesized finding 6:** Safety, accessibility, and aesthetics in the home were important	Category 6.1- Individuals value safety and accessibility for their homes
	Category 6.2- Individuals valued the aesthetics of their home
**Synthesized finding 7:** Family, community support and connection were essential to support remaining at home	Category 7.1- The importance of access to essential community services
	Category 7.2- The importance of being close or connected to family
	Category 7.3- Individuals felt a sense of connection towards their community

### Synthesized finding 1: People value independence, autonomy and housing that fits with their functional abilities

This synthesized finding comprised two categories as below in the Supplementary table 1, Additional file 5.

#### Category 1.1- Individuals perceived themselves as “independent” if they remained in their own home

Participants reported a strong desire to remain independent and in their own home. They believed that if they were “independent”, they would be able to remain in their home. They appeared to take pride in being independent in daily activities and not requiring help. Having choice over when and where to go was valued; being ‘forced’ into housing decisions made some participants feel trapped. In their daily lives, participants spoke of the importance of being able to choose what they did, where they spent their time and being in control of their own routines. Having a home with a garden was also described as giving the person a sense of freedom. Some participants did acknowledge that home modifications enabled them to remain independent at home.

“A home is your own, of course, and you can close your door and be by yourself when you want, and then it’s great fun to open up and be able to receive your friends and enjoy being at home” [43].

#### Category 1.2- The need for the “right fit” between an older person’s abilities and the environment

Participants acknowledged that as they aged their ability to manage different activities (such as home maintenance) declined. In some cases, changes in health status or ability resulted in the need to relocate. Homes that were well designed (such as having flat entry from the carport to the front door) made life easier as did homes that were specifically designed to be low maintenance. Participants were happy when there was a good fit between their abilities and the design of the home.

“I like the thought of having a garden that I'm in control of, rather than it being in control of me.” pg. 1187 [38]

### Synthesized finding 2: Finances and costs constrained decisions about future housing

This synthesized finding comprised one category as presented in the Supplementary material, table 2, Additional file 5.

#### Category 2.1- Financial resources were a factor when making housing decisions

Financial constraints impacted on people in a variety of ways. Some participants were in rental properties where the owners refused to fund home modifications which would have improved safety and access. Some participants felt that if they had to relocate, they had very few options (if any) that would be affordable. One person commented that they hadn’t expected to live as long as they were living. Factors such as leaving an inheritance for family members and deciding to invest in the housing market were also part of decision making.

“Wondering where I will be able to live when my money and health require another place . . .,” and “The economy has affected all of us. It’s harder and harder to survive financially . . . didn’t expect us to live as long as we’re living . . .” [44]

### Synthesized finding 3: People experienced feelings of stigma regarding ageing and were concerned about being a burden

This synthesized finding comprised four categories in the Supplementary table 3, Additional file 5.

#### Category 3.1- Negative views associated with moving house

Several participants indicated that they wanted to die in their own home and that they would rather die than move to a nursing home. Nursing homes were considered to have extensive rules and regulations (like a ‘prison’) where residents had poor quality of life. For some, this was tempered with a fear of being isolated and living (and dying) alone. Some participants acknowledged that their home possessed safety hazards but moving out of home would mean compromising on quality of life.

"Going into a home? That's be the end of me. And I mean it.” [45]

#### Category 3.2- Individuals did not want to be a burden

Concern about being a burden on others, and particularly family members, was a theme present in multiple participants and studies. Participants preferred to remain in their own homes rather than become a ‘burden’ to their family and others. Some participants indicated that their family had their own responsibilities and commitments and therefore it would be onerous for them to also provide care.

“I don't want to be a burden on my children, and I am willing to go to a nursing care facility, but my children say I should live with one of them.” [46]

#### Category 3.3- Stigma

For some, there was stigma associated with the installation of home modifications and relocation to residential care homes. The stigma associated with these objects or behaviours led to negative stereotypes of ageing and feelings of vulnerability among the participants. For instance, residential care homes and assisted living were considered to be an indication of loss of quality of life and dependency.

“When the ramp was finished, this workman with a really loud voice called out “this is now a disability house!” really loudly—the whole street would have heard.” [7]

#### Category 3.4- Acceptance of ageing impacted housing decisions

In contrast, participants who were more accepting of ageing, appeared to be more comfortable with the changes they experienced due to ageing. This acceptance enabled greater acceptance of home modifications and changed abilities. For some, acceptance enabled them to make proactive housing decisions.

‘‘I will accept being admitted to the nursing home when I need to go there – I hope.” [47]

### Synthesized finding 4: People experienced both positive and negative attitudes to future housing

This synthesized finding comprised two categories in the Supplementary table 4, Additional file 5.

#### Category 4.1: Individuals with positive attitudes to future housing

Many participants had considered steps they would need to take to ensure their future housing was age friendly. They spoke of seeking out or modifying their homes to ensure accessibility. Modifications and equipment were considered acceptable steps in terms of improving the age-friendliness of the home. Some participants mentioned that they would be willing to consider use of smart technologies (such as ambient assisted living) if it helped them to stay in their own home safely for longer. Others mentioned that they planned to move closer to family. Seeing others make housing decisions or speaking with others about their experience was considered helpful. One participant described how a move to residential care would be the best solution as they had limited family and would not be independent forever.

“I have already thought about it, if I can't manage to do things on my own anymore, then they should put me in a home. I don't have any children, and well, you have to be realistic, this would be the best solution.” [48]

#### Category 4.2: Some individuals avoid thinking about future housing

In contrast, there were older adults who chose to avoid or make no firm plans about future housing, including taking a ‘wait and see’ approach. Participants who felt this way were either unconcerned about the future, didn’t want to consider the future, or were unaware of steps that could be taken to safely age in place. One study described participants who were unsure of where, or how, to access home modification services.

“People actually don’t know that these services are out there. And also how to access them. You don’t get taught, at any point in your life, how to become an older person. It just sort of happens, [. . .] You know, if you have a child,... you’ve got your health visitor and they explain what you’re supposed to do. You become old and no-one is there telling you.” [49]

### Synthesized finding 5: Emotions, meaningful activities, and attachments to the home played a part in housing decisions

This synthesized finding comprised three categories in Supplementary table 5, Additional file 5.

#### Category 5.1: Emotions related to home

Several participants discussed how they achieved great satisfaction which stemmed from caring for their own home, garden and pets. Others spoke about how keeping busy within the home provided them with a reason to get up in the morning. Beyond the home, participants spoke about the sense of community they experienced and being able to trust others within their community. Home was described as a place of comfort where restoration occurred. Many participants described the feeling of being safe and secure in their own home and contrasted the freedom and privacy they experienced in their own home to the lack of privacy they would have in residential care homes. For some participants, home was described as place where they longed to escape due to marital breakdown or noisy neighbours. This experience exacerbated their desire to move sooner than later in life.

“The garden, front garden and looking up at the sky and the back garden with the lovely birds . . . just sitting at the kitchen table and looking out at the garden at the birds.” [33]

#### Category 5.2: Meaningful activities within the home

Being able to have the ‘space’ for meaningful activities was important. Participants mentioned activities such as gardening, using a backyard workshop for hobbies, an office and an area for art and crafts.

"See, we’ve got the front bedroom as our main bedroom, the second bedroom is Lara’s artist room, the third bedroom is my office . . . We’re using the whole house . . . It’s a seven-room house and we’re using them all.” [33]

#### Category 5.3: Strong sense of attachment to the home

Many participants felt strongly attached to their home and couldn’t imagine living elsewhere. They described wanting to remain in their own home until death in which case they would be leaving ‘in a box’. Participants, in some cases, were still living in the family home and were reluctant to leave. Home was filled with important possessions accumulated over their lifetime. The home and the possessions within often triggered memories; bringing feelings of joy and gratitude.

“Would rather leave in a box or we'll stay here till the last day I'm sure of it”. (pg. 1701 [50])

### Synthesized finding 6: Safety, accessibility, and aesthetics in the home were important

This synthesized finding comprised two categories in Supplementary table 6, Additional file 5.

#### Category 6.1: Individuals value safety and accessible homes

Many participants valued safety as an important feature of their home. Safety was linked to having flat, hazard free spaces or having modifications which improved access and safety. For example, stairs were seen as unsafe. Some participants wanted to be connected; either through being able to summons assistance in an emergency or having other people check in. Some participants mentioned that it was important to know and be able to trust their neighbours. One study described how night lights, safety guards and security systems contributed to a greater sense of safety.

“I wanted an apartment where I could feel safe, [a place where] if people want to come to my place they must buzz downstairs. Then I can answer directly and know that my door is locked. But it’s not everywhere you can find a building with this type of security. I will stay there for a long time!” (pg.365 [51])

#### Category 6.2: Individuals valued the aesthetics of their home

Natural, bright lighting and open spaces appealed to middle aged and older adults. They spoke of how a house feels like a home depending on the decorations and possessions within. Personal possessions and mementos added to the appeal of the home. While home modifications were sometimes viewed as improving the appearance of the house, more commonly they were seen as visually unappealing and contributed to the house feeling like a clinical environment instead of a home.

“With the best will in the world, adaptations can, you know, provide a very clinical... a more clinical environment, as assessed by need. And it’s trying to have that.... That, sort of, conversation with them about what is in their best interest, really. To keep them in the home, safe. And I think you have to be very sensitive to that. (pg.7 [49])

### Synthesized finding 7: Family, community support and connection were essential to support remaining at home

This synthesized finding comprised three categories in the Supplementary table 7, Additional file 5.

#### Category 7.1: The importance of access to essential community services

Health care services, alternative housing options, meals and home cleaning were all essential services deemed to be important to support older adults to remain in their own home. It was further highlighted that these needed to be culturally appropriate services. One participant mentioned the lack of housing options that can meet older people’s housing needs. Some older adults identified the importance of having good access to public transport nearby their homes as this linked them beyond their own home and was especially needed after cessation of driving.

“Everybody worries about it (transportation). My neighbor across the street is 77 and she might not be able to drive in a couple of years, and she says 'as soon as I can't drive, I've got to move.” (pg. 153 [52])

#### Category 7.2: The importance of being close or connected to family

Participants felt it was important for their homes to be nearby family. Being close to others within their social networks strengthened connection to family, prevented isolation and provided them with regular ‘check-in visits’. Families helped with practical tasks such as transport and home maintenance in order to help them remain in their own homes. In some cases, children of ageing adults were involved in housing decisions (for example, inviting older relatives to live with them or moving closer). Participants valued having family visit or stay with them and enjoyed using their home to entertain. Otherwise, one participant felt dwindling visits from family and friends made home more isolating.

“When she (neighbour) look up (from her apartment) she can see me. When she notices I do not open my window she will telephone me. I advised my son (adopted son in law), 'you call me on the phone in the morning and in the evening. If something happens to me at night, you will know in the morning. This way it is fine. If something happens to me in the day, when you call at night, you will also know' ... I fell very at ease.” (pg. 530 [53])

#### Category 7.3: Individuals felt a sense of connection towards their community

Older adults felt strongly connected to their communities. Family and friends often lived nearby, and, in some cases, participants considered their neighbours to be like family or friends. In that way, the community contributed towards the person’s social network. Furthermore, they appreciated knowing the people who worked in the local shops. The person’s community contributed towards their sense of identity. Participants mentioned the value of having community services, local amenities, and public transport nearby as well as green spaces open to public use. One participant voiced concerns about segregating middle aged and older adults from younger communities (such as in retirement villages or communities).

“I have very good friends. I’ve been widowed since 1978, and had I not had those friends, it would have been very difficult for me. And then they’re like family … very close good friends that care about you.” (pg. 774 [54])

## Discussion

This review included the findings of qualitative research studies regarding the perspectives of middle aged and older adults of their home environments and identified several key themes. Factors deemed important when making decisions about future housing were: (a) independence, abilities, and autonomy; (b) finances and costs; (c) feelings of stigma regarding ageing and concerns about being a burden; (d) positive and negative attitudes to ageing; (e) emotions, meaningful activities, and attachments with the home; (f) safety, accessibility, and aesthetics in the home; (g) family, community support and connection whilst remaining at home. Having an understanding of these perspectives allows professionals working in home and community design, health and social care to support ageing in place [55]. These factors should be considered when planning and designing communities which support ageing adults. This study synthesizes the views of middle aged and older adults from an international context (from Australia to Finland) to understand future housing decision-making internationally.

This systematic review of qualitative studies and meta-aggregation builds on the findings of individual studies and provides up- to-date evidence regarding middle aged and older adult’s experience of their home. Systematic reviews advance knowledge through identifying and analysing multiple studies, identifying gaps in the literature, understanding deficiencies in current studies and helping to guide delivery of care and policy development [56]. We identified a total of 46 studies published over the last 15 years demonstrating the strong interest and importance of this topic area. We were interested in the views of middle-aged adults as they may be considering their future needs when planning renovations, relocation and/or downsizing. However, only twelve of the studies included people considered to be middle aged (as part of a larger participant group including older people) and their views were not presented separately. More research should be conducted specifically with this population group as decisions made during this time may lead to long term benefits. For example, someone who is 50 years old and renovating their bathroom could ensure that there is flat entry to the shower alcove and could avoid installing a low toilet seat.

Older adults tended to have negative views related to relocation and residential care; this finding was not unexpected. Consistent with the findings from, Gillsjö, Schwartz-Barcott [57] and Corcoran, Bernard [58], most middle aged and older adults want to remain in their homes for as long as possible. It is possible that perceptions of living in residential care are currently poor due to media reports in the last few years portraying residential care as being an environment of neglect [59]. Work is needed in the residential care sector to demonstrate that high quality care can be offered and that autonomy can be preserved. A recent integrative review regarding autonomy in residential care showed that autonomy was a critical contributor to health and quality of life. Moilanen, Kangasniemi [60] also found that autonomy could be preserved in residential care, but this depended on staff skill and family support. Simple things such as being listened to and decorating one’s own room contributed to autonomy [61].

It is also evident that people become more reluctant to consider moving as they age and moving appears to be more difficult as time passes. Despite peoples’ intentions to remain at home for as long as possible there are currently a number of barriers in place that need to be addressed at a policy level. In terms of care, more investment in home care services is required. For example, data from 2021 showed that older Australians spent an average of 28 months on a wait list for home care [62]. Other research suggests, home care packages for people in small communities in Portugal are in limited supply [63]. Similarly, whilst individuals within Europe such as England, Austria and France have access to a large variety of home care services, long-term funding continues to be a problem [63]. Therefore, by the time middle aged and older adults gain access to funding for care, they will receive less care than they need and face the risk of further decline, preventable hospitalisation, and premature entry to residential aged care [59]. Furthermore, dedicated funding for home modifications is not currently available. Our review showed that financial constraints play a part in decision making around future housing; new solutions are required so that older adults can access funds for home modification without needing to use their home care services budget.

Aside from the physical features of the home, this review showed similar results to Tanner, Tilse [7] that many participants described their home as a place of security, autonomy, and comfort. These emotions are not easily created and continue to take time to develop in their homes. As adults age, homes trigger these emotions and begin to tie into everyday routines [64]. Similar to studies by Sherman and Dacher [18] and Oswald, Wahl [65], those who wanted to stay in their home became emotionally attached to their property. Moving therefore is not simply a transaction in space and planners and designers need to look beyond the physical barriers at home, but also their individual meaning of home. This confirms findings by Coleman and Wiles [21] regarding the relationship between ageing, the physical environment, and personal views about ageing.

The ageing population’s changing demographics will continue to create demand for affordable age-friendly housing. The affordability of age friendly housing is an important consideration for planners and policy makers. In America, Li, Hu [66] suggested the need to increase rental assistance funding for ageing adults to promote affordable housing. Possible solutions include, either densifying housing units to build smaller units or transforming single-family houses such as garages and basements, into smaller housing units [66]. Similarly, Riedy, Wynne [67] suggested that co-housing may have the potential to address the challenges older adults face with regards to affordability, accessibility and isolation. However, their research also showed there were negative perceptions of cohousing amongst the ageing population, due to the lack of familiarity with shared living arrangements [67]. Jolanki [68] recommends the need for more ‘in-between’ housing options for all stages of ageing and housing policies to meet the rapid growth of older adults.

Results also agreed with studies by Stones and Gullifer [8], Kramer and Pfaffenbach [9], Hatcher, Chang [10], where living near family, friends and acquaintances was described as being important. Feelings of loneliness and social isolation is common in older people and is expected to increase as the ageing population increases [69]. Depending on the country the estimates of social isolation and loneliness can vary. Literature indicates between 12 to 30% experience loneliness and between 5 to 17% of older people are socially isolated [70–72]. Older adults experience a decline in economic and social resources, continued functional limitations and changes in family structure [73]. Social isolation and loneliness can place older people in greater risk of mortality and comorbidities [74]. To address this, the World Health Organisation [74] recommends connecting older adults to services and maintaining/building relationships. Remaining socially connected to others who live nearby enables ageing adults to feel safer and less anxious [75]. Luciano, Pascale [76] developed a framework for age-friendly housing including nine domains, which includes ‘community connection’. Hence, living near family, friends and neighbours are an important aspect of ensuring older adults are not socially isolated, but a part of a locally integrated network.

### Strengths and limitations

This review showed the importance of consulting with middle aged and older adults and understanding their perspectives when planning communities and designing housing for older adults. The included studies contained rich information from a diverse range of journals, however, the review is subject to some limitations. It is possible that as the topic was so broad some relevant studies may have been missed using the selected search strategies. This review also did not source non-English studies, lacking the perspectives of middle aged and older adults from countries where English is not the primary language. Though qualitative methods assisted in the understanding of perceptions about home among middle aged and older adults, we were not able to understand which characteristics were most important. For example, participants may have spoken about the importance of living near family however in real life this may be less important than other features, such as housing affordability. Another limitation is that the experiences of participants may have been influenced by other factors, such as cognitive impairment or other chronic conditions.

## Conclusion

In summary, this review provides a guide to assist with consideration of future housing needs that should incorporate middle aged and older adult’s values around their home, rather than focusing only on the physical characteristics of the home. Working with middle aged and older adults to develop age friendly communities and buildings may promote autonomy and independence, reduce isolation and loneliness and result in people staying at home for longer which also results in reduced government spending. Changes to funding are required so that older adults can access funding specifically for appropriate home modification. Given that it may not always be possible to stay at home, alternatives to residential care (such as co-housing) should be trialled. Older adults and their families who are contemplating relocation should consider how the move can be a positive experience through ensuring new housing feels safe and aesthetically pleasing. They should also consider how the person can maintain connection to their previous community while forming connections with their new community.

### Supplementary Information

**Additional file 1.** Prisma 2020 Checklist.

**Additional file 2.** Search strategies.

**Additional file 3.** Characteristics of included studies.

**Additional file 4.** Characteristics of studies.

**Additional file 5.** Summary of synthesised findings and cateogories. **Table S1.** Synthesized finding 1 - Independence, abilities, and autonomy. **Table S2.** Synthesized finding 2- Finances and costs constrained decisions about housing. **Table S3.** Synthesized finding 3- Feelings of stigma regarding ageing and concern about being a burden. **Table S4.** Synthesized finding 4- Positive and negative attitudes to future housing. **Table S5.** Synthesized finding 5- Emotions, meaningful activities, and attachments with the home. **Table S6.** Synthesized finding 6- Safety, accessibility, and aesthetics in the home. **Table S7.** Synthesized finding 7- Family, community support whilst remaining at home.

## Data Availability

The datasets generated and/or analysed during the current study are available in the Flinders University research management repository, https://doi.org/10.25957/pk90-9092.
