# Supplementary material for: Middle aged and older adult’s perspectives of their own home environment: a review of qualitative studies and meta-synthesis
Source: BMC Geriatr. 2023 Oct 31;23:707. doi: 10.1186/s12877-023-04279-1 (PMC10619279; doi:10.1186/s12877-023-04279-1)
Supplement: Supplementary file 2 — Additional file 2. Search strategies. [file 12877_2023_4279_MOESM2_ESM.docx]

# Additional file 2: Search strategies

## Medline, search conducted May 11, 2021 and July 12, 2022

| **#** | **Searches** | **Records retrieved** |
| --- | --- | --- |
| 1 | aged/ or "aged, 80 and over"/ or frail elderly/ | 3406338 |
| 2 | (elder* or geriatric* or gerontolog* or old age* or grandparent* or retire* or pensioner* or senior* or later life).tw. | 431814 |
| 3 | ((old* or age* or aging) adj1 (person* or people* or adult* or resident* or population* or men* or women* or male* or female*)).tw. | 636866 |
| 4 | (aged adj1 ("65" or "70" or "75" or "80" or "85")).tw. | 50588 |
| 5 | or/1-4 | 3929212 |
| 6 | independent living/ | 9946 |
| 7 | ("aging in place" or "ageing in place" or "aging-in-place" or "ageing-in-place" or "age in place" or "aging at home" or "ageing at home" or "aging-at-home" or "ageing-at-home" or living independently or independent* living or living autonomously or autonomous living or "living at home" or "remaining at home" or "residing at home" or retirement communit* or "communit* for retirement" or "home environment*").tw,kw. | 16056 |
| 8 | ((physical or environment*) adj2 (space or limit* or challang*)).tw,kw. | 13719 |
| 9 | or/6-8 | 38227 |
| 10 | interviews as topic/ or focus groups/ or narration/ or qualitative research/ | 155357 |
| 11 | ((semi-structured or semistructured or unstructured or informal or "in-depth" or indepth or "face-to-face" or structured or guide? or group*) adj3 (discussion* or questionnaire*)).tw. | 59965 |
| 12 | (Interview* or focus group* or diary or diaries or transcrib* or verbatim or field not* or memo? or memoing).tw. | 533378 |
| 13 | (audiotap* or audio-tap* or audio record* or audiorecord* or tape record* or taperecord* or video*).tw. | 169793 |
| 14 | ((context* or semantic or content) adj2 analys*).tw. | 44937 |
| 15 | (narrat* or qualitative* or ethnograph* or fieldwork or field work or field research* or informant* or phenomenolog* or hermeneutic* or grounded or interpretive* or participant observ* or background observ* or reflective* or reflection* or textual* or open-ended or theme? or thematic* or triangulat*).tw. | 652499 |
| 16 | ((personal* or patient* or participant* or lived) adj2 (experience or experiences or perception* or perceptive or perspective*)).tw. | 112736 |
| 17 | or/10-16 | 1293890 |
| 18 | 5 and 9 and 17 | 4297 |

## PsycINFO (Ovid), search conducted May 11, 2021 andJuly 12, 2022

| **#** | **Searches** | **Records retrieved** |
| --- | --- | --- |
| 1 | older adulthood/ | 10552 |
| 2 | (elder* or geriatric* or gerontolog* or old age* or grandparent* or retire* or pensioner* or senior* or later life).tw. | 153951 |
| 3 | ((old* or age* or aging) adj1 (person* or people* or adult* or resident* or population* or men* or women* or male* or female*)).tw. | 230213 |
| 4 | (aged adj1 ("65" or "70" or "75" or "80" or "85")).tw. | 14233 |
| 5 | or/1-4 | 343352 |
| 6 | ("aging in place" or "ageing in place" or "aging-in-place" or "ageing-in-place" or "age in place" or "aging at home" or "ageing at home" or "aging-at-home" or "ageing-at-home" or living independently or independent* living or living autonomously or autonomous living or "living at home" or "remaining at home" or "residing at home" or retirement communit* or "communit* for retirement" or "home environment*").tw,id. | 13493 |
| 7 | ((physical or environment*) adj2 (space or limit* or challang*)).tw,id. | 3968 |
| 8 | or/6-7 | 17396 |
| 9 | qualitative methods/ or exp focus group/ or grounded theory/ or interpretative phenomenological analysis/ or narrative analysis/ or semi-structured interview/ | 18437 |
| 10 | ((semi-structured or semistructured or unstructured or informal or "in-depth" or indepth or "face-to-face" or structured or guide? or group*) adj3 (discussion* or questionnaire*)).tw. | 30141 |
| 11 | (Interview* or focus group* or diary or diaries or transcrib* or verbatim or field not* or memo? or memoing).tw. | 409666 |
| 12 | (audiotap* or audio-tap* or audio record* or audiorecord* or tape record* or taperecord* or video*).tw. | 87038 |
| 13 | ((context* or semantic or content) adj2 analys*).tw. | 36052 |
| 14 | (narrat* or qualitative* or ethnograph* or fieldwork or field work or field research* or informant* or phenomenolog* or hermeneutic* or grounded or interpretive* or participant observ* or background observ* or reflective* or reflection* or textual* or open-ended or theme? or thematic* or triangulat*).tw. | 548875 |
| 15 | ((personal* or patient* or participant* or lived) adj2 (experience or experiences or perception* or perceptive or perspective*)).tw. | 75621 |
| 16 | or/9-15 | 894142 |
| 17 | 5 and 8 and 16 | 1567 |

## CINAHL, search conducted May 11, 2021 and July 12, 2022

| # | Searches | Records retrieved |
| --- | --- | --- |
| S18 | S5 AND S16 AND S17 | 6,118 |
| S17 | S6 OR S7 OR S8 | 34,200 |
| S16 | S9 OR S10 OR S11 OR S12 OR S13 OR S14 OR S15 | 674,090 |
| S15 | TI ( ((personal* OR patient* OR participant* OR lived) N1 (experience OR experiences OR perception* OR perceptive OR perspective*)) ) OR AB ( ((personal* OR patient* OR participant* OR lived) N1 (experience OR experiences OR perception* OR perceptive OR perspective*)) ) | 65,411 |
| S14 | TI ( (narrat* OR qualitative* OR ethnograph* OR fieldwork OR "field work " OR "field research* " OR informant* OR phenomenolog* OR hermeneutic* OR grounded OR interpretive* OR "participant observ* " OR background observ* OR reflective* OR reflection* OR textual* OR "open-ended " OR theme* OR thematic* OR triangulat*) ) OR AB ( (narrat* OR qualitative* OR ethnograph* OR fieldwork OR "field work " OR "field research* " OR informant* OR phenomenolog* OR hermeneutic* OR grounded OR interpretive* OR "participant observ* " OR background observ* OR reflective* OR reflection* OR textual* OR "open-ended " OR theme* OR thematic* OR triangulat*) ) | 327,112 |
| S13 | TI ( ((context* OR semantic OR content) N1 analys*) ) OR AB ( ((context* OR semantic OR content) N1 analys*) ) | 32,932 |
| S12 | TI ( (audiotap* OR "audio-tap* " OR "audio record* " OR audiorecord* OR "tape record* " OR taperecord* OR video*) ) OR AB ( (audiotap* OR "audio-tap* " OR "audio record* " OR audiorecord* OR "tape record* " OR taperecord* OR video*) ) | 62,973 |
| S11 | TI ( (Interview* OR "focus group* " OR diary OR diaries OR transcrib* OR verbatim OR "field not*" OR memo* OR memoing) ) OR AB ( (Interview* OR "focus group* " OR diary OR diaries OR transcrib* OR verbatim OR "field not* " OR memo* OR memoing) ) | 354,253 |
| S10 | TI ( ((semi-structured OR semistructured OR unstructured OR informal OR "in-depth" OR indepth OR "face-to-face" OR structured OR guide? OR group*) N2 (discussion* OR questionnaire*)) ) OR AB ( ((semi-structured OR semistructured OR unstructured OR informal OR "in-depth" OR indepth OR "face-to-face" OR structured OR guide? OR group*) N2 (discussion* OR questionnaire*)) ) | 30,675 |
| S9 | (MH "Qualitative Studies+") | 170,793 |
| S8 | TI ( ((physical OR environment*) N1 (space OR limit* OR challang*)) ) OR AB ( ((physical OR environment*) N1 (space OR limit* OR challang*)) ) | 4,144 |
| S7 | TI ( ("aging in place" OR "ageing in place" OR "aging-in-place" OR "ageing-in-place" OR "age in place" OR "aging at home" OR "ageing at home" OR "aging-at-home" OR "ageing-at-home" OR "living independently" OR "independent* living" OR "living autonomously" OR "autonomous living" OR "living at home" OR "remaining at home" OR "residing at home" OR "retirement communit*" OR "communit* for retirement" OR "home environment*") ) OR AB ( ("aging in place" OR "ageing in place" OR "aging-in-place" OR "ageing-in-place" OR "age in place" OR "aging at home" OR "ageing at home" OR "aging-at-home" OR "ageing-at-home" OR "living independently" OR "independent* living" OR "living autonomously" OR "autonomous living" OR "living at home" OR "remaining at home" OR "residing at home" OR "retirement communit*" OR "communit* for retirement" OR "home environment*") ) | 10,773 |
| S6 | (MH "Community Living") OR (MH "Assisted Living") | 21,597 |
| S5 | S1 OR S2 OR S3 OR S4 | 1,546,461 |
| S4 | TI ( (aged N0 ("65" OR "70" OR "75" OR "80" OR "85")) ) OR AB ( (aged N0 ("65" OR "70" OR "75" OR "80" OR "85")) ) | 21,041 |
| S3 | TI ( ((old* OR age* OR aging) N0 (person* OR people* OR adult* OR resident* OR population* OR men* OR women* OR male* OR female*)) ) OR AB ( ((old* OR age* OR aging) N0 (person* OR people* OR adult* OR resident* OR population* OR men* OR women* OR male* OR female*)) ) | 220,927 |
| S2 | TI ( elder* OR geriatric* OR gerontolog* OR "old age*" OR grandparent* OR retire* OR pensioner* OR senior* OR "later life" ) OR AB ( elder* OR geriatric* OR gerontolog* OR "old age*" OR grandparent* OR retire* OR pensioner* OR senior* OR "later life" ) | 189,281 |
| S1 | (MH "Middle Age") OR (MH "Frail Elderly") OR (MH "Aged, Hospitalized") OR (MH "Aged, 80 and Over") OR (MH "Aged") | 1,409,450 |

## Scopus (Elsevier), search conducted May 12, 2021 and July 12, 2022

| # | Search | Records retrieved |
| --- | --- | --- |
| S1 | ( ( TITLE-ABS-KEY ( ( elder* OR geriatric* OR gerontolog* OR old AND age* OR grandparent* OR retire* OR pensioner* OR senior* OR later AND life ) ) OR TITLE-ABS-KEY ( ( ( old* OR age* OR aging ) W/0 ( person* OR people* OR adult* OR resident* OR population* OR men* OR women* OR male* OR female* ) ) ) OR TITLE-ABS-KEY ( ( aged W/0 ( "65" OR "70" OR "75" OR "80" OR "85" ) ) ) ) ) AND ( ( TITLE-ABS-KEY ( ( "aging in place" OR "ageing in place" OR "ageing-in-place" OR "age in place" OR "aging at home" OR "ageing at home" OR "aging-at-home" OR "ageing-at-home" OR "living independently" OR "living autonomously" OR "living at home" OR "remaining at home" OR "residing at home" OR "retirement communit*" OR "communit* for retirement" OR "home environment*" ) ) OR TITLE-ABS-KEY ( ( ( physical OR environment* ) W/1 ( space OR limit* OR challang* ) ) ) ) ) AND ( ( TITLE-ABS-KEY ( ( ( semistructured OR unstructured OR informal OR "in-depth" OR indepth OR "face-to-face" OR structured OR guide* OR group* ) W/2 ( discussion* OR questionnaire* ) ) ) OR TITLE-ABS-KEY ( ( interview* OR focus AND group* OR diary OR diaries OR transcrib* OR verbatim OR field AND not* OR memo* ) ) OR TITLE-ABS-KEY ( ( audiotap* OR audio-tap* OR audio AND record* OR tape AND record* OR video* ) ) OR TITLE-ABS-KEY ( ( ( context* OR semantic OR content ) W/1 analys* ) ) OR TITLE-ABS-KEY ( ( narrat* OR qualitative* OR ethnograph* OR fieldwork OR field AND work OR field AND research* OR informant* OR phenomenolog* OR hermeneutic* OR grounded OR interpretive* OR "participant observ*" OR "background observ*" OR reflective* OR reflection* OR textual* OR open-ended OR theme* OR thematic* OR triangulat* ) ) OR TITLE-ABS-KEY ( ( ( personal* OR patient* OR participant* OR lived ) W/1 ( experience OR experiences OR perception* OR perceptive OR perspective* ) ) ) ) ) | 1620 |
