# Supplementary material for: Middle aged and older adult’s perspectives of their own home environment: a review of qualitative studies and meta-synthesis
Source: BMC Geriatr. 2023 Oct 31;23:707. doi: 10.1186/s12877-023-04279-1 (PMC10619279; doi:10.1186/s12877-023-04279-1)
Supplement: Supplementary file 3 — Additional file 3. Characteristics of included studies. [file 12877_2023_4279_MOESM3_ESM.docx]

# Additional file 3: Characteristics of included studies

| **Study** | **Phenomena of interest** | **Participant characteristics and sample size** | **Methods for data collection and analysis** | **Description of main results** |
| --- | --- | --- | --- | --- |
| Almevall et al. (2022), Sweden | To describe experiences of home from a well-being perspective, describe participant characteristics and well-being measures in relation to housing type, and how the aforementioned aspects may affect well-being in very old persons | n= 50  Age: 80 years or older living in ordinary housing were interviewed (July 2017 to November 2018) about home in relation to well-being, along with collection of participant characteristics and well-bring measures related to home.  20 men and 30 women, ages 81-96 years living in ordinary housing were interviewed  Middle aged and/or older adults included: Only older adults. | Methodology: Cross-sectional, convergent parallel-results mixed methods design  Methods:  Data collection-  Semi-structured interviews. Specific questions for the current study regarding well-being in relation to home were posed. Interview questions were open-ended and consisted of two areas: what are the most positive aspects of living in your home, and do you find that there are any difficulties involved with living in your home?  Data analysis: qualitative content analysis, data saturation was reached in the sense that the information would be enough to replicate the study. Microsoft Office Excel 2013 was used for structuring coding and categorization. | Six subcategories, three categories and one theme describing experiences of home in relation to well-bring among very old persons.   For some, home was described as a place of loneliness and isolation. Ageing in place as a political vision might be misdirected if it fails to take consideration the complexity and nuances of home and what these means for well-being in very old persons.   Rather than focusing on preventing older people from moving into specialised housing or offering alternative living adapted with only built environment in focus, society must offer this group living conditions and support that maintain their sense of well-being. |
| Aplin et al. (2013), Australia | To explore the impact of home modifications on clients and their family’s experience of home | n= 55  Age: 25-87 years, living in a metropolitan area in Australia, with an average age of 64 ranging from 25 to 87 years.  They had lived in their homes on average for 18 years, ranging from six months to 62 years.  Middle-aged and/or older adults included: Both included. Number of middle-aged adults not stated. | Methodology: Qualitative descriptive approach   Methods:  Data collection- in-depth interview using a semi-structured questionnaire in their own home. Interviews were audio recorded and transcribed verbatim.  Data analysis: Template analysis was selected as this form of qualitative descriptive analysis. | Home is linked to the occupant's identity. reflects who they are both positive and negative effects. Positive appearance of modified area was enhanced by the modification. New modern bathroom, kitchen or front access was often enjoyed.  Freedom was negative impacted or not restored for a small number of participants as she was not provided with the modification that enabled access to her garden or outdoor area. Carer roles and activities became easier as a result of modifications. |
| Aplin et al. (2015), Australia | To explore the impact of home modifications on clients and their family’s experience of home | n= 55  Age: 64-87 years, clients, spouses, family and carers of the service, living in metropolitan area in Australia.  42 household interviews included 13 joint interviews  Middle-aged and/or older adults included: Only older adults | Methodology: Qualitative descriptive approach   Methods:  Data collection- one in-depth interview using a semi-structured questionnaire in their own home. Interviews were audio recorded and transcribed verbatim  Data analysis- Template analysis was selected as this form of qualitative descriptive analysis. | Home is linked to the occupant's identity. reflects who they are- both positive and negative effects. Positive- appearance of modified area was enhanced by the modification. New modern bathroom, kitchen or front access was often enjoyed. - participants were disappointed with the appearance of modifications, feeling like they made their home look disabled or like a hospital. Some did not worry about appearance of modifications as they then valued their home as practical and functional places - modifications allowed people to regain or maintain independence and freedom in the home. |
| Bailey et al. (2019), Australia | Experiences of 30 older people acquiring and living with home adaptations and on the findings from four focus group discussions. | n=30 older people and 39 practitioners  Age: 65–74, 75–84, and 85+ . Over six months, participants’ lived experiences were captured within this two-strand approach.  Middle-aged and/or older adults included: Older adults only. | Methodology: phenomenological approach  Methods:  Data collection-Strand 1: semi-structured interviews carried out with older adults in their own homes Strand 2: practitioner focus group discussions with a total of 39 practitioners, involved two focus group discussions at each of the two participating local authority sites.  Data analysis: interviews and focus groups discussions were transcribed verbatim and entered into NVivo. | An overarching finding raised by older adults and practitioners, that delaying having home adaptations until a person was “struggling”, was partly to do with perceived negative associations of ageing, with loss of independence and vulnerability and because of their medicalised appearance. 1. Ageism and home adaptations; participants spoke about triggers signalling disability and clinical aesthetics (appearance and utility). Practitioners talk of how when they see older adults they are not aware that there is something wrong or need at their age. Word of mouth raises awareness of adaptations- older people might have experience of a friend or neighbour who has specific equipment. |
| Baron et al. (2020), Canada | The aim was to explore the perspective Inuit elders on the relationships between aging, health and place. | The community of Baker Lake was selected for the study because of its provision of several housing units dedicated to adults over 60 years old, as well as the presence of a long-term care centre for Inuit with chronic conditions or disabilities. twenty Inuit aged between 50 to 86, from one community in Nunavut, participated to in-depth qualitative interviews.  Middle aged and/or older adults included: Both included. Number of middle-aged adults not stated. | Methodology:  Qualitative study  Methods:  Data collection- in-depth Interviews and the focus group  Data analysis: Thematic content | Themes discussed included aging and health, housing conditions, community conditions, land-based activities, medical and leisure travel outside of the community, and mobility and accessibility. Four main groups of resources for healthy aging were identified by participants during individual interviews and the focus group: 1) family relationships; 2) adequate housing conditions; 3) positive community conditions, and; 4) being on the land. |
| Bergland and Slettebø (2018), Norway | To explore how older Norwegian women living at home experience ageing, and how their everyday life has been influenced by their encounters with the challenges of life | N=10 women aged 90 years or older living in their own home in Norway  Middle-aged and/or older adults included: Only older adults. | Methodology:  qualitative design  Methods:  Data collection- semi-structured interviews  Data analysis- qualitative content analysis in line with Kvale and Brinkmann’s analysis of interview transcripts | The overall theme for the findings is how everyday life in old age is influenced by past, present and future. Participants found it difficult to make plans in old age Participants accepted that their lives had to become what they are now .Acceptance of life as it has become: Acceptance of relocation later in life.. |
| Bigonnesse et al. (2014), Canada | An overview presents how older adults express meaning of home and what their needs are regarding housing and relocation | n= 392 (49 focus groups) and one case study from the Age-Friendly Cities Project in Quebec.  Age: 65–74 years and 75 years and older. Income levels (low and mid).  Middle-aged and/or older adults included: Only older adults. | Methodology:  qualitative methodology  Methods: Data collection-in-depth interviews Data analysis- Thematic analysis of these 2 sources of qualitative data and open coding | Three subthemes emerged from the analysis under the theme built environment: (a) affordable housing and services, which covers issues around affordability of housing resources and home-based services; (b) adapted and adaptable homes, which covers home adaptation and home modification; and (c) safe and good quality homes, which covers the safety and suitability of current housing resource), (b) neighborhood, (c) community, and (d) privacy |
| Black et al. (2015), America | To better understand how older adults, and the broader community, perceive and promote dignity and independence in the context of everyday community life. | n= 484 persons participated in the study including the community-based forums and online participation (n = 217) and qualitative inquiry (n = 261) approaches including seven focus groups and e-surveys. 3 different samples of participants for the qualitative inquiry that included community forums (n = 113), seven focus groups (51 participants), and online surveys (n = 320).  Age: 65 or older  Middle-aged and/or older adults included: Only older adults. | Methodology: PAR and multiple methods of qualitative inquiry . Grounded Theory  Methods:  Data collection-focus groups and open-ended surveys with semi structured interview guide for the focus group.  Data analysis- grounded theory | Synthesized findings yielded 6 “actionable themes”: (1) meaningful involvement, (2) aging in place, (3) respect and inclusion, (4) communication and information, (5) transportation and mobility, and (6) health and well-being. |
| Bosch-Farre et al. (2020), Catalonia, Spain | To explore the ageing in place phenomenon, as well as the enablers and barriers that interact in a healthy ageing from the perspective of the elderly connected to local entities. | n=71  Age: men and women aged 65 years who can speak Catalan or Spanish, age ranged 62 to 92 years.  Regarding gender, 60.56% were women and 39.43% were men.  Middle-aged and/or older adults included: Only older adults. | Methodology:  Qualitative design  Methods:  Data collection-6 focus groups, semi-structured guide.  Data analysis- Inductive and deductive thematic analysis was employed | Three key themes were generated:  1. Participants experienced ageing differently. The physical and mental health, the family environment and financial stability were key elements for life quality. 2. The perception of the elderly’s role in the community depended on their age, health status and attitude towards life.  3. The participants identified several enablers and barriers to healthy ageing in place. Home was described as a place to feel free, comfort and enjoyment of your own privacy. Living in place is an important factor of connexion and social inclusion, especially if people go out and engage with entities of social support, such as in the case of the present study. |
| Brim et al. (2021), Philadelphia, America | This project seeks to narrow this literature gap and explore perceived barriers reported by older adults who are aging in place. | n= 36  Age: 55 years living in the Mt. Airy, Chestnut Hill, Germantown, and Northwest Philadelphia neighbourhoods  Middle-aged and/or older adults included: Both included. Number of middle-aged adults not stated. | Methodology: grounded theory  Methods:  Data collection-Review of reports from older adult home safety assessments. Assessments were scheduled by the older adult via phone or email. Semi-structured interview followed by an older adult-guided tour through the home.  Data analysis: Constant comparison with existing data form the last 4 years. | Home mobility and safety were recognised by older adults as current or potential hazards to moving safely in their home. Older adults reported fall hazards as barriers towards ageing in place. Lighting within the home was a common barrier for ageing in place. Clutter or junk within the home interfered with function within an older person's home. Safety devices within the bathroom was needed to age in place. |
| Burgess and Quinio (2021), England | To explore why older households, move and why they choose either new-build housing or existing stock | n=13  Age: 55 or over and has moved less than a year ago  Middle-aged and/pr older adults included: Both included. 44% were aged between 55 and 64. Number of middle-aged adults not stated. | Methodology: Mixed methods   Methods: English Housing Survey (EHS), two online surveys to collect primary data.  Data analysis: Qualitative analysis | Only a minority of them actually choose to downsize. Older people's narratives and experiences confirmed that their decisions to move were often driven by a varierty of factors, circumstances and aspirations which characterize a heterogeneous group of older people |
| Coleman and Wiles (2020), New Zealand | How the possessions that older adults keep at home inform their present experiences of aging and also facilitate the ongoing maintenance of aging in place | n= 28 (20 women, 8 men)  Age: 65-94. Four were aged between 90-94 years old. 11 participants returned journals with annotated comments.  Middle-aged and/or older adults included: Only older adults. | Methodology: Phenomenological inspired research  Methods:  Data collection- in-depth interviews, photo elicitations interviews and journaling  Data analysis: interpretive approach | 3 main experiences from the participants.  1. Being surrounded and maintaining insideness.  2. Being with past achievements and maintaining self.  3. Being self-reflective, connected and reconciling All 3 factors play a role in the experience of aging in place and in being able to maintain daily life during ageing.  Cherished possessions fostered connection and belonging (other words, an insideness of place), and a routine way of perceiving aging in place and the character of everyday experiences. |
| Dahlin-Ivanoff et al. (2007), Sweden | to explore and broaden understanding of home very old persons experience the meaning of home and how the concept of home was related to autonomy, well-bring and participation. | n= 40 (17men and 23 women)  Age= 80-89  All participants lived in ordinary housing, majority lived in apartments, living in single-family housing. Lived in their apartment from 1-81 years.  Middle-aged and/or older adults included: Only older adults. | Methodology: grounded theory as per of ENABLE-AGE project. ENABLE-AGE survey study  Methods:  Data collection- interviews and field notes taken.  Data analysis: constant comparative analysis | Home is a central place in the lives of very old people because it is where they live and spend so much time. Home means security and freedom. Home means security, living in a familiar neighbourhood, everything functions, and having memories to live on. Home means freedom comprises a place for reflection, a social-meeting point and leaving your own mark. Home has a central place within the lives of older people. Living in a familiar neighbourhood- home means being close to someone in case one needs help, which creates feeling of safety and security. Having memories, being surrounded by people who have their memories and by the people who have been there. eg. photos |
| de Jonge et al. (2011), Australia | This study reports on the experiences of older people in Australia of their home environments. Explores and describes the role of the home environment in older people's lives. | n=30 older people with diverse characteristics Sample recruited from 3 states, QLD, SA and Vic.  Age: 56-90  7 men, 23 women and 16 living alone, 11/14 were couples that participated in the interview together.  Middle-aged and/or older adults included: Both included. 2/30 participants were middle-aged. Aged 56 and 60. | Methodology: Phenomenological approach  Methods:  Data collection- Semi-structured interviews. Open-ended questions. Audio taped and transcribed verbatim.  Data analysis: content analysis | Five themes from 30 transcripts. 1. Capacity of the home to support valued roles and meaningful occupations.  2. Independence and autonomy Home gave a sense of freedom, autonomy, remaining in the home was central towards independence  3. Social connectedness Older people were happy with their current home as it provided connection with community services eg. transport, shops, bowls club, GP and hydro pools. Being around family and neighbours were important  4. Ambience of the home and lifestyle afforded.  5. History and emotional connection with the home. against an intruder. |
| Dendle K, Miller E, Buys L, Vine D. 2021, Australia | To explore how a range of diverse older adults across Australia experience their homes | n=103 Age: 50-92  Included those aged 50+ because decisions affecting housing and their subjective effects are often influenced by life-stage (e.g. empty nest, retirement) and changes of circumstance (e.g. divorce, health changes) which may occur prior to an arbitrary age.  Middle-aged and/or older adults included: Both included.  Aged 50-59: 30 participants Aged 60-69: 26 participants  Aged 70-79: 34 participants  Aged 80+: 13 participants | Methodology:  Qualitative phenomenographic framework   Methods:  Data collection- Focus groups which ran over a 4-day period, in conjunction with a private online focus group  Data analysis: Experiences of home identified using Nvivo 12. Further manual analysis and discussion with co-authors grouped into 'conceptions' | Older people saw their home as the centre of their wider world. Their home extended beyond the bounds, through the yard or grounds, and further to public, private and virtual spaces outside the private dwelling that the literature usually considers as 'home'. Greater focus is needed from policymakers and practitioners on 'home' as a combination of place, space and community unique to each person. More adaptable housing options is needed through housing policy to enable people with varied access to resources to optimise the match between needs aspiration. |
| Dupuis-Blanchard et al. (2015), Canada | To gain a better understanding of ageing in place by exploring how older adults who experience a loss of independence and speak a minority language (French are able to remain in their homes, despite the challenges they face | n=39 older adults residing at home but experiencing a loss of independence. 14 family members, 10 family members were relative of older participants in the study and 4 relatives of an older person with loss of independence who resided at home.  Age: Average age of the older participants was 81 years, ranging from 65-93.  Middle-aged and/or older adults included: Only older adults. | Methodology: qualitative descriptive study  Methods:  Data collection-Semi structured interviews  Data analysis: Content analysis, verbatim transcriptions were analysed line by line using open coding. | 6 themes emerged-health consciousness, housing choice, access to services, social support, and income. Older people explained they live in a home which met their needs, outdoor maintenance and cleaning were presented by older people as challenging tasks like lawn mowing, cleaning, painting, washing floors Home was a place of security. Older people, inclu family members attributed apartment living to feeling of security. |
| Elo et al. (2011), Finland | The purpose of this study was to construct a theory on an environment that would support the well-being of home-dwelling elderly people in northern Finland. | n= Phase 1 consisted of 39 interviews of elderly people over the age of 65. Phase 2 consisted of 96 postal questionnaires and 3 expert evaluations. 15 panel evaluations. Phase 3 consisted of 328 postal questionnaires.  Middle-aged and/or older adults included: Only older adults. | Methodology: Used both qualitative and quantitative methods  Methods:  Data collection- focus, postal questionnaires and interviews. Inductive concept synthesis, hypothetical models. Second phase was evaluated by a panel of experts consisting of 15 nurses by postal questionnaire.  Data analysis: principal component analysis and confirmatory factor analysis | An environment that enables safe activity and a pleasant physical environment was important for the elderly. Climate and availability of services are related to the well-being of the elderly. An environment that enables safe activity comprises both safety at home and immediate surroundings that enable safe mobility. At home, aspects including ensuring safety of stairs and steps, reducing the need to reach or climb using various support rails, making floors and the bathroom less slippery. |
| Fausset et al. (2009), Georgia, America | How older adults cope with their problems and suggested areas for redesign to improve older adults' daily lives Investigate and to understand how older adults are managing and maintaining their homes, to inform designers (architects) and industrial designers how to support these tasks. | n= 20 (13 women and 7 men). Eligible participants were required to live independently and not reside in any type of housing with home maintance support.  Age: 73-85  Middle-aged and/or older adults included: Only older adults. | Methodology: Focus group method  Methods:  Data collection- focus groups were conducted with 6 to 7 individuals. Groups were divided by sex, martial status and race. Telephone presreening that assessed basic cognitive functioning, background questionaire, technology questionaire and exit survey.  Data analysis: detailed coding scheme to identify patterns and themes from the discussions. | Home maintenance is required to ensure a safe and healthy environment. By understanding how older adults are managing and maintaining their homes, human factors can inform architects, home remodelers and redesigners about the areas of greatest need for older adults. Designers can enable aging in place and support older adults' desire to live in their home as long as want. |
| Finlay et al. (2020), America | Aimed to characterise salient features of built and social environments that are essential to support low-income ageing residents. | 38 individuals who lived in subsidised housing and homeless shelters.  Older participants were aged 55-92 in 3 distinct socio-economic and geographic samples of the Minneapolis metropolitan area. Nearly all participants lived alone, in their usual residential area by Downtown Minneapolis and Eden Prairie.  Middle-aged and/or older adults included: Both included.  Aged 55-91: 20 participants  Aged 55-91: 8 participants  Aged 67-92: 10 participants | Methodology:  Qualitative study   Methods:  Data collection- Seated and mobile interviews. Interviews with semi-structured questions. Transcripts, photographs and field notes  Data analysis: qualitative thematic analysis | Four themes encompassed essential residential qualities 1. safety and comfort 2. service access 3. social connection 4. stimulation Home was not necessarily an extension of the self. Ageing in place produced hazardous situations for some with sub-standard housing and lack of appropriate support. identity. |
| Fjell et al. (2021), Norway | To investigate how old persons perceived their life to be, how they viewed the ageing process and their need of health care and societal support | n=34  Age= 69-93 in 7 group discussions (28 women and 6 men). All resided in large municipality Inclusion: retired persons residing in their private home, can read, write and speak in Norweigan.  Middle-aged and/or older adults included: Only older adults. | Methodology: qualitative approach applying an explorative design  Methods:  Data collection-Focus groups to collect data via semi structured interviews  Data analysis: Inductive manifest content analysis | 3 categories- embracing life, dealing with challenges, considering the future, followed by sub-categories and the overall themes. Overall Themes “So Far so Good” with meaning that growing old was to enjoy life here and now. Considering the future, older people felt comfortable with the thought of moving to a residential home as they did not want to help from the home care services. Some older people wish to remain at home as long as possible provided they were given access to home care services. Older people want to move to shared accommodations with facilities that contribute to feeling secure and at the same time facilitate their social life. |
| Gould et al. (2017), Canada | To explore the thinking processes involved in planning or failing to plan for the future. To observe how older adults spontaneously address issues of future planning when not constrained to do so. | n= 39 interviews with older adults who were experiencing disability and illness but who lived in their own home. (30 women and 9 men)  Age: 65-93 (M=81)  Middle-aged and/or older adults included: Only older adults. | Methodology:  qualitative study  Methods:  Data collection- Structured interviews.  Data analysis: qualitative data analysis Interviews | Three approaches to thinking about the future 1. One day at a time 2. reasons not to go (into a nursing home) 3. If/then two under lying contextual factors  a) the influence of family members b) the availability of resources (financial and otherwise) exerted a subtle yet important influence on which approach to thinking about the future was used and how these approaches were used. The importance of physical spaces for family reunions to take place in senior's apartments may be important when designing facilities. Lack of planning leads to crises with particularly negative outcomes. |
| Grimmer et al. (2015), Australia | To explore and synthesize the experiences and perspectives of older people planning for and experiencing aging in place. | n= 42  Age: 65 and over (8 were >80 years)  Middle-aged and/or older adults included: Only older adults. | Methodology:  Qualitative study  Methods:  Data collection-  Semi-structured interviews and focus groups, audio-recorded and transcribed  Data analysis- Thematic analysis | 8 key elements for successful aging in place: health, information, practical assistance, finance, activity (physical and mental), company (family, friends, neighbours, pets), transport, and safety. All participants wanted to stay in the community home of their choice as long as they could, whether this was a larger family home or smaller independent living option. |
| Hatcher et al. (2019), New South Wales, Australia | To understand the phenomenon of older people living at home in Australia at a personal level, from the perspective both of those in living in their long-term family home and those have adjusted to newer living conditions in older age. | n= 21, recruited from a local government seniors centre in Western Sydney, Australia.  Age: 65 years or over , a resident of Western Sydney, NSW, Aus, currently living in their home for at least 12 months before study, english speaking and consented to study.  Middle-aged and/or older adults included: Only older adults. | Methodology:  Grounded theory  Methods:  Data collection- focus group discussions and in-depth semi structured interviews..  Data analysis- Open coding was conducted line by line on printed transcripts using the Strauss and Corbin or Microanalysis. | 4 main major categories were developed 1. Anchoring self 2. Enabling freedom 3. Being comfortable 4. Staying in touch Home represented their past, present, and future and gave the notion of anchoring self. |
| Juvani et al. (2005), Finland | The purpose was to describe the significance of the physical environment in supporting the well-being of the elderly aged over 65 year. | n= 39  Age: 65-89, from Northern Finland, dwelling at home, to be able to communicate verbally, and to have no serious memory deficits. (13 men, 26 women)  Middle-aged and/or older adults included: Only older adults. | Methodology: Qualitative study  Methods:  Data collection: Interviews  Data analysis: inductive content analysis | The northern physical environment seem to be significant for both urban and rural elderly people living in the northern part of Finland. The different aspects of the natural environment provided opportunities to relax, meet other people and do physical exercise, all of which are important factors of health promotion. |
| Lewis and Buffel (2020), United Kingdom | Understanding of the interrelationship between aging in place and the places of aging, revealing how these processes change over time. | n= 24   Age: 50 years and over  Middle-aged and/or older adults included: Both included. Number of middle-aged adults not stated. | Methodology:  qualitative longitudinal study  Method:  Data collection- longitudinal interviews  Data analysis: trajectory analysis’ approach | Living in a familiar environment has been identified as a crucial factor in the desire to remain at home for older people. Aging in place policies assume that the home and surrounding neighbourgood will remain familiar and predictable for older people. The longitudinal analysis has revealed how place attachment changes over time and can be highly unpredictable. |
| Mackenzie et al. (2015), Australia | To explore a relatively large sample of older people's subjective experiences, their expectations and the suitability of their home and neighbourhoods. | n= 202  Age: 75-79 years  This age group are considered most likely to still be in general health and at the stage of life where they may be considering a future move to a supportive home and environment.  Middle-aged and/or older adults included: Only older adults. | Methodology: qualitative study  Methods:  Data collection- semi-structured interviews  Data analysis- Thematic analysis | 6 key themes emerged from the interviews namely housing choice, attachement to place, financial issues, changes to the home over time, transport and anticipating the future. Home is both concrete and physical accomplishment. Home is intimately related to attachment, physical, social and autobiographical insideness, security and familiarity. |
| Martin et al. (2019), America | The purpose of this research is to examine the intent behind decisions to not age in place as means of informing future initiatives. | n= 1680 adults completed an anonymous Aging in Place Needs Assessment survey between Jan 2015 and Dec 2017.  Age: 65 and over  Middle-aged and/or older adults included: Only older adults. | Methodology: qualitative study  Methods:  Data collection- Surveys  Data analysis: qualitative coding of narrative surveys | 4 themes; need to downsize/home modification; need for assistance; family desire to reciprocate care; and isolation. Relocation often results in loss of social relationships or personal possessions, changes in daily routines and lifestyles and the eventual loss of independence. |
| Mortenson et al. (2016), Canada | To explore the how would surveillance technologies change the way older people experience the home environment? | n= 27  Age: 60 and over and had 1 self-reported chronic condition or mobility restriction.  Middle-aged and/or older adults included: Only older adults. | Methodology: qualitative study  Data collection-in-depth interviews  Data analysis: Interviews were transcribed verbatim and analyzed with QSR International’s NVivo 8 software. Unstated type of analysis. | 3 main themes, 'safe and sound' described how participants felt (ambient assisted living) AAL could contribute to their sense of security, 'reliance' explored how AAL would effect residents' autonomy, self-confidence and relationship with caregivers and 'under the the microscope' revealed how AAL-mediated surveillance might alter perceptions of home and activity participation. |
| Narushima and Kawabata (2020), Ontario, Canada | Explores the experience of aging among older Canadian women with physical limitations who live by themselves. | n= 12 women living in 2 geographic areas in Southern Ontario, including residents of regular houses, apartments, condominiums, assisted living and community housing for seniors. All in varying states of health  Age: 65-92  Middle-aged and/or older adults included: Only older adults. | Methodology:  qualitative study  Data collection- interviews  Data analysis- thematic analysis | 4 overarching themes 1. Striving to continue on at home 2. Living as a strong independent woman 3. The help needed to support their 'independence' 4. Social activities to maintain self participants 'independent' life styles were supported by many other people in a mix of formal and informal care. |
| Neville et al. (2021), Rural New Zealand | To explore the views of older people about their preparation for ageing well in a rural community. | n=49  Age: 65-93 31 females and 19 males interviewed, 15 were widowed, 23 were married, 2 were single and 10 divorced. Lived independently in the study area and accessed the local service town for goods and services  Middle-aged and/or older adults included: Only older adults. | Methods: Qualitative study using interpretive description.  Methodology:  Data collection-purposive sampling. Semi-structured in-depth interviews were, lasting between 45 and 90 minutes.   Data analysis: Thematic approach. Deductive or theory-driven approach. Six phases of thematic analysis as outlined by Braun and Clarke. | 3 main themes identified: 1) sensible planning: the right place and the right people; 2) remaining independent: its up to me; and 3) facing challenges: accepting my lot. All age groups were actively and realistically preparing for ageing well. All valued independence and believed in the importance of planning for their future. Policy makers and practitioners need to understand that older people are a heterogenous group ageing policies should be geared towards' older people's individual abilities and circumstances. |
| Neville et al. (2016), Auckland, New Zealand | To explore how individuals aged 95 years and older living in their own home remain socially connected. | n= 10 (8 women and 2 men)  Age: 96-100 years  6 people lived in small town houses and 4 lived in family-sized houses with gardens. 9 people lived on their own and 1 person identified herself as being the primary caregiver for her son who has a disability.  Middle-aged and/or older adults included: Only older adults. | Methodology: Qualitative design  Methods:  Data collection-semi-structured interviews  Data analysis: thematic analysis | 3 main themes illuminating social connectedness were identified, 'keeping company: staying connected with family and friends', 'doing things together: engaging with paid and unpaid helpers', and 'having pride and enjoyment: continuing with hobbies and interests'. |
| Norazizan et al. (2006), Serdang, Malaysia | To describe the difficulties faced by older Malaysians in their present home environment. | n= 386, randomly selected across urban areas in Malaysia, residing in 5 urban locations were interviewed, mostly pertaining to factors associated with problems faced in their home. Respondents chosen were generally in good health with no obvious disabilities.  Age: 60 > years  Middle-aged and/or older adults included: Only older adults. | Methodology: not stated  Methods:  Data collection- Interviews  Data analysis-not stated  . | Ageing-in-place is one of the highest priorities of the elderly, and those who wish to do so should be enabled to remain in their own home as long as possible. An ergonomic approach to its designing would improve the relationship between the elderly user and his/her environment, thus encouraging ageing-in-place. |
| Nosraty et al. (2015), Finland | To discover how 90-91 year old see a good old age and identify the dimensions of good and successful aging that appear in their talk | n= 45 (25 women and 20 men)  Age: 90> years  Middle-aged and/or older adults included: Only older adults. | Methodology: qualitative study  Methods: Data collection- interviews and short questionnaires.  Data analysis- Thematic analysis with an inductive approach | Theories of successful aging such as the physical, the cognitive, the psychological and social functioning. New themes were 'living circumstances', emphasizing the importance of having one's own home and living there as long as possible. 'independent' in relation to various aspects of life and a 'good health'. |
| Owens et al. (2021), South Carolina, America | To document the lived experiences among older low-income African Americans who live alone. | n= 12, (5 men, 7 women) Fluent English speakers, lived alone, no self-reported cognitive or physical impairment preventing them from participating in the study.  Age: 66-80  Middle-aged and/or older adults included: Only older adults. | Methodology: Ethnography  Data collection- Surveys, engaged in video diary recording and participated in in-depth follow-up interviews.  Data analysis-thematic analysis  thematic categories developing larger narrative themes. Transcripts using Nvivo. | Findings supported the the person-environment fir model interplay of belonging and agency, aging well, and the environment model which predicts that perceptions of belonging in an environment grow stronger among older adults as they age and their functional impairment declines. Compensatory strategies for overcoming barriers to person-environment included home modifications such as installation of grab bars in the bathroom. |
| Park and Ko (2020), South Korea, Korea | To explore the sociocultural meaning of 'my place' for elderly Koreans ahead of enacting a policy for the so-called 'integrated community care' in South Korea, pursuant to aging in place for the elderly population. | n= 10  Age: 65 >  Middle-aged and/or older adults included: Only older adults. | Methodology: Ethnography  Data collection- in-depth interviews, field notes  Data analysis-Spradley's ethnographic approach | 3 categories representing the meaning of 'my place' were emerged, 1. keeping me safe and 2. comfortable, representing my life, and 3. maintaining my control and influence. |
| Puplampu et al. (2020), Canada | To examine the impact of cohousing on older adults' quality of life. Research question, "what is the impact of seniors' cohousing on older adults' quality of life? | n= 23  Age: 65> years  Middle-aged and/or older adults included: Only older adults. | Methodology: Mixed methods  Data collection-self-administered surveys, individual interviews, focus groups  Data analysis- Thematic analysis | Four themes of 'belonging in a community', 'life in the community', changes associated with aging, and 'aging in place' emerged from the qualitative data to explain factors that influence older adults' quality of life. Belonging in a community describes participants' satisfactions with their quality of life in the building. Older adults in the cohousing community indicated that 1 of the reasons for choosing cohousing was because they wanted to be in a living arrangement where they felt that they belonged; they cared about each other; and they were engaged and felt safe. |
| Renaut et al. (2015), France | To understand how individuals construct the space both within their own home and their immediate surroundings and how this construction is linked to their own perception of ageing and growing old. | n= 28 older people and 17 carers  Age: 75> years and carers 45> years providing care to someone above 65  Middle-aged and/or older adults included: Only older adults. | Methodology:  qualitative study  Methods:  Data collection- semi-directive questionnaire, photographs  Data analysis-Thematic analysis | Old age was perceived as an inevitable decline based on the experience of their ageing parents and had begun to adapt their home environment in anticipation of poor mobility, disability and gradual illness. Hazards such as carpeting, steep stairs without banisters or cluttered living spaces were unnoticed by some frail older participants aged 80 and above. Participants would only act when the time was right due to confidence that they would have some resources to do so even if decisions would be hard to make. Participants only chose to act when the time was right despite age, the notion of prevention. |
| Shin et al. (2021), Wisconsin, America | To explore older adults' daily interactions with their home environments. | n= 23, who all received home modifications to successfully age in place. (13 female, 10 male)  Age: 62-89  Middle-aged and/or older adults included: Only older adults. | Methodology: qualitative study  Methods:  Data collection- 3D scanning and biomarker tracking technology with qualitative in-depth qualitative interviews.  Data analysis Conventional content analysis used | Most common strategy was to modify their behaviours by limiting or giving up certain activities (cooking, taking a shower, going out). Environmental adaptations and changes were frequently discussed with participants however they were too concerned about the high cost. Inaccessible homes pose significant barriers to participants, however modifying them became a delicate conundrum. The home modification industry is highly fragmented between health care and home assessment professionals and handyman services. |
| Sixsmith et al. (2014), Great Britain | To examine the ways in which very old people perceive healthy ageing in the context of living alone at home within urban settings in five European Countries (Germany, Hungary, Latvia, Sweden and the United Kingdom. | n=190 people, (117 women, 73 men), all participants were living at home in urban settings.  Age: 75-89  Middle-aged and/or older adults included: Only older adults. | Methodology:  qualitative study  Methods:  Data collection- in-depth and semi-structured interviews  Data analysis- grounded theory approach | The physicality and spatiality of home provided the context for establishing and evaluating the notion of healthy ageing, whilst the experienced relationship between home, life history and identity created a meaningful space within which healthy ageing was negotiated. By being able to manage at home gave older people a sense of existence. Older adults expressed the importance of maintaining a sense of control and independence in the home. Depending on how older adults understood home, their attitudes of healthy ageing and their ability to maintain active lifestyles differed. |
| Tan et al. (2015), Finland | To explore older people's experiences of living independently or with an unrelated older person. | n= 25, in Singapore, lived alone with spouse or with an unrelated older person, and were able to communicate in Mandarin, English or Chinese dialects (Cantonese or Hokkien).  Age: 65-95  Middle-aged and/or older adults included: Only older adults. | Methodology:  descriptive qualitative study  Data collection-face to face interviews with open ended questions  Data analysis-thematic analysis | 5 themes emerged 1. Making own choice, participants decided to live apart from their families. 2. Contending with concerns, the availability of external resources for participants was shrinking. 3. Coping with the available assistance, depending on available external resources from the community. 4. Holding on their values, participants rely on their internal resources to manage.  5. Preparing for the inevitable, participants were planning for their final years of life and for their death. |
| Tanner et al. (2008), Australia | What is the impact of home modification on the experience and meaning of home to older people living in the community? | n= 12, (4 men and 8 women) had received home modification in the previous 3 to 6 months were chosen from occupational therapy reports in public housing files to include a range of ages, living arrangement, level of disability, and gender, housing types, and home modifications. Mixture of people living alone (n = 5), living with a partner (n = 5), or with other family (n = 2).  Age: 65> living in public housing in a metropolitan area and has had home modifications installed to their current accommodation within the previous 3-6 months.  Middle-aged and/or older adults included: Only older adults. | Methodology: Qualitative methodology-   Methods:  Data collection- Audiotapes of the interviews were transcribed verbatim into written data, including observed responses, other researcher notes and memos.  Data analysis: N-VIVO software was used for data and thematic analysis of the transcripts. | Major themes discussed:  1. What participants said about their experience and the meaning of home Personal Home- making meaning through action - participants 'made' their house into a home created more of a personal meaning. 2. How modifications affected this meaning -Modifications strengthened the personal home as a place of security, safety, comfory and control - Modifications have increased independence, safety, comfort in performing daily tasks.  3. Effect of service design and delivery on the the experience and meaning of home for older people -Less satisfaction on the design as they were concerned about the implication of designing for wheelchair accessibility. |
| Vrkljan et al. (2011), Canada | To explore the relationship between habitual occupations and environmental features that can support aging in place. | n= 10  Age: 65> years, lived alone or were married and had no caregivers coming into the home to support their daily activities.  Middle-aged and/or older adults included: Only older adults. | Methodology: Phenomenological approach  Data collection-Respective interviews.  Data analysis- Open coding process described by Hsieh and Shannon | Habitual occupations provided a vital link their community. Participants felt the determination to maintain their level of participation by adapting the way in which they engaged in certain habits. Participants continually highlighted the important of maintaining regular occupations to keeping them going and enabling them to stay healthy and engaged in their community. |
| Webber et al. (2022), United Kingdom | How homes are made and unmade over time and to look at how experiences of home are dynamically shaped by people's potent connections inside and outside the dwelling. | n=28  Age: 50> lived in one of the 4 chosen neighbourhoods.  Middle-aged and/or older adults included: Both included. Stated ages of 3 middle-aged adults, aged 58, 61 and 57. | Methodology: Qualitative longitudinal study  Methods:  Data Collection- Interviews and transcribed   Data analysis: NVivo, with a coding framework | The experience of home changed in different ways, particularly through their activities and interests within their dwellings and in the wider community. Home unmaking was more prevalent due to their dwindling social ties, decreased engagement in activities beyond the home, and the deteriorations of their physical and mental health, resulting in their sense of home becoming less porous and more isolating over time. A sense of home strengthens over time. A sense of belonging to home must be understood in the context belonging to place, relationships, routines, and familiarity with place. |
| Wiles et al. (2012), Auckland, New Zealand | To discuss with older people what aging in place meant to them and whether it necessarily meant staying in the same place and advantages or disadvantages of that. | n= 121  Age: 56-92 (44 men, 77 women)  Middle-aged and/or older adults included: Both included. Number of middle-aged adults not stated. | Methodology: qualitative study  Methods:  Data collection- interviews in 2 case study communities, focus groups  Data analysis- thematic and narrative analysis | Older people want choice about where and how they age in place. Aging in place was seen as an advantage in terms of a sense of attachment or connection and feelings of security and familiarity in relation to both homes and communities. Aging in place related to a sense of identity both through independence and autonomy and through caring relationships and roles in the places people live. Aging in place had a practical advantage for the security and safety of home. It was important for participants to in their homes as long as possible for familiarity. |
| Woolrych et al. (2020), India, Brazil, United Kingdom | How place insideness is experienced amongst older adults across India, Brazil, and the United Kingdom | n=294  Age: 60-94, either residing in the neighbourhoods of India, Brazil and United Kingdom.  Middle-aged and/or older adults included: Both included. Number of middle-aged adults not stated. | Methodology: Qualitative research methods   Methods:  Data Collection- Face to face semi-structured interviews collected across 9 cities and 27 neighbourhoods, go-along interviews, and photo diaries.  Data analysis: Cross-national analysis. | Lower-income communities in India and Brazil was notable different from the United Kingdom, including the absence of basic physical infrastructure which created barriers to accessing key services and opportunities.  Place identity is being threatened within areas of urban regeneration and rapid physical transformation which is undermining a sense of connection to place with communities. |
| Yu and Rosenberg (2017), Beijing, China | To explain the spatio-temporal processes of older people with their changing places. To explain how old age and place work in the context of urban China. | n= 47 (23 men, 51 women)  Age: 60> years, currently residing at home in the community, with Beijing Hukou (Household registrar system).  Middle-aged and/or older adults included: Both included.  Aged 60-69: 9 participants  Aged 70-79: 20 participants Aged 80+: 18 participants | Methodology:  qualitative study  Methods:  Data collection- semi structured interviews with open ended questions  Data analysis- Comparative methods | Older people interpreted the meanings of place and their identities in the changing environments. The past are present in current place identity construction and why among the older people, thus how they cope in society in transition and their understanding of aging in place. Current older generation, their place identities are deeply rooted in pre reform collectivism and shaped by pre-reform socialist ideologies (equality, standardisation, uniformity). |
