# Supplementary material for: Middle aged and older adult’s perspectives of their own home environment: a review of qualitative studies and meta-synthesis
Source: BMC Geriatr. 2023 Oct 31;23:707. doi: 10.1186/s12877-023-04279-1 (PMC10619279; doi:10.1186/s12877-023-04279-1)
Supplement: Supplementary file 4 — Additional file 4. Characteristics of studies. [file 12877_2023_4279_MOESM4_ESM.docx]

# Additional file 4: Characteristics of studies

| **Citation** | **Q1** | **Q2** | **Q3** | **Q4** | **Q5** | **Q6** | **Q7** | **Q8** | **Q9** | **Q10** |
| --- | --- | --- | --- | --- | --- | --- | --- | --- | --- | --- |
| Almevall et al. (2022) | Y | Y | Y | Y | Y | Y | Y | Y | Y | Y |
| Aplin et al. (2013) | U | Y | Y | Y | Y | N | N | Y | Y | Y |
| Aplin et al. (2015) | U | Y | Y | Y | Y | U | N | Y | Y | Y |
| Bailey et al. (2019) | U | U | Y | Y | Y | N | N | Y | Y | Y |
| Baron et al. (2020) | U | U | U | U | U | N | Y | Y | Y | Y |
| Bergland and Slettebø (2018) | Y | Y | Y | Y | Y | Y | Y | Y | Y | Y |
| Bigonnesse et al. (2014) | U | U | Y | Y | Y | N | U | Y | Y | Y |
| Black et al. (2015) | Y | Y | Y | Y | Y | N | Y | Y | U | Y |
| Bosch-Farre et al. (2020) | Y | Y | Y | Y | Y | N | Y | Y | Y | Y |
| Brim et al. (2021) | U | Y | Y | Y | Y | N | N | N | Y | U |
| Burgess and Quinio (2021) | Y | U | Y | U | Y | Y | N | Y | N | Y |
| Coleman and Wiles (2020) | Y | Y | Y | Y | Y | N | N | Y | U | Y |
| Dahlin-Ivanoff et al. (2007) | U | Y | Y | Y | Y | N | U | U | Y | Y |
| de Jonge et al. (2011) | U | Y | Y | U | Y | N | N | Y | Y | Y |
| Dendle et al. (2021) | Y | Y | Y | Y | Y | Y | Y | Y | Y | Y |
| Dupuis-Blanchard et al. (2015) | U | Y | Y | U | Y | U | U | N | Y | Y |
| Elo et al. (2011) | U | Y | Y | U | U | N | U | N | U | U |
| Fausset et al. (2009) | U | Y | Y | U | U | N | N | N | U | U |
| Finlay et al. (2020) | U | Y | Y | Y | Y | N | N | Y | Y | Y |
| Fjell et al. (2021) | Y | Y | Y | Y | Y | Y | U | U | Y | Y |
| Gould et al. (2017) | U | Y | Y | Y | Y | N | N | Y | Y | Y |
| Grimmer et al. (2015) | Y | Y | Y | Y | Y | Y | Y | Y | Y | Y |
| Hatcher et al. (2019) | U | Y | Y | Y | Y | N | N | Y | Y | Y |
| Juvani et al. (2005) | U | Y | Y | Y | Y | N | N | Y | U | Y |
| Lewis and Buffel (2020) | U | Y | Y | Y | Y | Y | N | Y | U | Y |
| Mackenzie et al. (2015) | Y | Y | Y | Y | Y | N | N | Y | Y | Y |
| Martin et al. (2019) | U | Y | N | Y | Y | N | N | U | Y | Y |
| Mortenson et al. (2016) | Y | Y | Y | Y | Y | N | N | Y | U | Y |
| Narushima and Kawabata (2020) | U | Y | Y | Y | Y | N | N | Y | Y | Y |
| Neville et al. (2021) | U | Y | Y | Y | Y | Y | U | Y | Y | Y |
| Neville et al. (2016) | Y | Y | Y | Y | Y | Y | N | Y | Y | Y |
| Norazizan et al. (2006) | Y | Y | Y | U | Y | Y | U | N | N | Y |
| Nosraty et al. (2015) | U | Y | Y | Y | Y | N | N | Y | Y | Y |
| Owens et al. (2021) | U | U | U | U | U | N | N | Y | Y | Y |
| Park and Ko (2020) | U | Y | Y | Y | Y | Y | N | Y | Y | Y |
| Puplampu et al. (2020) | U | Y | Y | Y | Y | N | Y | Y | Y | Y |
| Renaut et al. (2015) | U | Y | Y | Y | Y | N | N | Y | U | Y |
| Shin et al. (2021) | U | U | U | U | U | N | N | N | Y | U |
| Sixsmith et al. (2014) | U | Y | Y | Y | Y | N | N | Y | Y | Y |
| Tan et al. (2015) | U | Y | Y | Y | Y | N | N | Y | Y | Y |
| Tanner et al. (2008) | Y | Y | Y | Y | Y | N | N | Y | Y | Y |
| Vrkljan et al. (2011) | Y | Y | Y | Y | Y | N | Y | Y | Y | Y |
| Webber et al. (2022) | U | U | Y | U | U | N | N | N | N | Y |
| Wiles et al. (2012) | U | Y | Y | Y | Y | N | N | Y | Y | Y |
| Woolrych et al. (2020) | U | Y | Y | Y | Y | Y | U | Y | Y | Y |
| Yu and Rosenberg (2017) | U | Y | Y | Y | Y | N | N | Y | Y | Y |
| % | 32.6 | 84.78 | 91.3 | 78.26 | 86.95 | 26.08 | 19.56 | 78.26 | 76.08 | 91.3 |
| **Y: yes; N: no; U: unclear; JBI Critical Appraisal Checklist for Qualitative Research**  **Q1 = Is there congruity between the stated philosophical perspective and the research methodology?**  **Q2 = Is there congruity between the research methodology and the research question or objectives?**  **Q3 = Is there congruity between the research methodology and the methods used to collect data?**  **Q4 = Is there congruity between the research methodology and the representation and analysis of data?**  **Q5 = Is there congruity between the research methodology and the interpretation of results?**  **Q6 = Is there a statement locating the researcher culturally or theoretically?**  **Q7 = Is the influence of the researcher on the research, and vice-versa, addressed?**  **Q8 = Are participants, and their voices, adequately represented?**  **Q9 = Is the research ethical according to current criteria or, for recent studies, is there evidence of ethical approval by an appropriate body?**  **Q10 = Do the conclusions drawn in the research report flow from the analysis, or interpretation, of the data?** | | | | | | | | | | |
