# Supplementary material for: Middle aged and older adult’s perspectives of their own home environment: a review of qualitative studies and meta-synthesis
Source: BMC Geriatr. 2023 Oct 31;23:707. doi: 10.1186/s12877-023-04279-1 (PMC10619279; doi:10.1186/s12877-023-04279-1)
Supplement: Supplementary file 5 — Additional file 5. Summary of synthesised findings and cateogories. Table S1. Synthesized finding 1 - Independence, abilities, and autonomy. Table S2. Synthesized finding 2- Finances and costs constrained decisions about housing. Table S3. Synthesized finding 3- Feelings of stigma regarding ageing and concern about being a burden. Table S4. Synthesized finding 4- Positive and negative attitudes to future housing. Table S5. Synthesized finding 5- Emotions, meaningful activities, and attachments with the home. Table S6. Synthesized finding 6- Safety, accessibility, and aesthetics in the home. Table S7. Synthesized finding 7- Family, community support whilst remaining at home. [file 12877_2023_4279_MOESM5_ESM.docx]

# Additional file 5: Summary of synthesised findings and cateogories

## Table 1: Synthesized finding 1 - Independence, abilities, and autonomy

Abbreviation: (U) Unequivocal finding, (C) credible finding

| Finding | Illustration |
| --- | --- |
| Category 1.1- Individuals perceived themselves to be independent if they remained in their own home | |
| Freedom facilitated autonomy and control in their planning of everyday (U) | "I'm used to being on my own and when you're on your own you can do what you like, you can eat what you like, you can go to bed and get up when you feel like it... I like to be able to do things my way. If you've got me living with anyone they say well you do so and so and they might do it different to what I would do it.. I feel happy living in my own home ...Doing what I want to do and doing it when I feel like doing it."^pg. 5(Hatcher et al., 2019)^ |
| The sense of self-reliance was highly associated with their own homes (U) | “I feel free and ... I am still able to do the things (that) need to be done and the things I want to do.” ^pg. 108(Mortenson et al., 2016)^ |
| Home is a meeting-place where everything happens, and a place family or friends can be received into (U) | “A home is your own, of course, and you can close your door and be by yourself when you want, and then it’s great fun to open up and be able to receive your friends and enjoy being at home.” ^pg. 29(Dahlin-Ivanoff et al., 2007)^ |
| Older adults stress the importance of freedom of choice about the moment they relocate (U) | “When you lose your autonomy, then the game is different. Sometimes you don’t have the choice anymore. If you wait until the last minute [to relocate], you can’t choose for yourself. The kids will decide for you, and you will be admitted for the best or the worst.”^pg.375(Bigonnesse et al., 2014)^ |
| Being socially connected with others out of home activities was important (U) | “Mondays are shopping day. We go out and shop somewhere. She bowls on Monday afternoons after we shop. Tuesday, after swimming, Tuesday is usually her day to go out with the girls, so I’ll stay home while she goes out with the girls, so I'll stay home while she goes out. Wednesday we go swimming again and usually Wednesday afternoon we meet friends. Either go out for lunch or even just go out for a coffee. We met all these people at the pool. . . they aren't old friends. They are people we met at the pool- now we're all friends.”^pg. 154(Vrkljan et al., 2011)^ |
| Living independently provided freedom to perform everyday living tasks which may not be possible if they relocated (U) | "I do all my own work."^pg. 4(Hatcher et al., 2019)^ |
| Changes were made to retain independence (U) | "I could get more help if I needed it, but I said no that'll do for the moment.” ^pg. 1806(Grimmer et al., 2015)^ |
| Being able to make their own choices was important in being independent. (U) | “We don’t like to go into these retirement villages that they’re trying to get everybody into today Int: Why is that? C: We like our own space, you know, and like to be independent. We had friends who moved into one out [that] way and they’ve got a nice little two bedroom place, but they can’t do anything to the gardens. Everything is done. They had a name on the door of their old house and they wouldn’t let them put that up there. And you can’t have animals. . . . . Some people are quite happy to have organised things around them and that, you know, like these friends of mine, that’s why they have fitted in so well. They have little concerts up in the hall . . . the gardens are done and everything . . . [but] we still like gardening, we always did and we always swore we wouldn’t get a place unless it had a little bit of dirt, not a big bit, but just a little bit for therapy, you know.” ^pg. 363(Wiles et al., 2012)^ |
| Remaining in the home was central towards independence (U) | “I’ll fight till the last for that (to stay here).” ^pg. 43(de Jonge et al., 2011)^ |
| There was a strong desire to remain in their own home due to its link to independence and autonomy. (U) | “That’s independence too, isn’t it? It just sort of makes you more able to do things [general agreement.” ^pg. 362(Wiles et al., 2012)^ |
| Being able to live in one’s own home is enough to constitute successful ageing (U) | “A good old age is this, like mine, this is good. It makes me sick when I watch the telly, showing old people in those institutions. And they always say they're not doing well. And it just makes me ill to see that, but my situation is good.”^pg. 55(Nosraty et al., 2015)^ |
| Not everyone wants to live in cohousing communities (U) | “I mean, it's not an option for everybody, probably for relatively a small proportion of the population, which is too bad. Having to think about somebody other than your own, your own immediate household.” ^pg. 412(Puplampu et al., 2020)^ |
| They felt trapped if they were unable to move in a place (U) | “It means i'm stuck, I can't move (laughter).” ^pg. 360(Wiles et al., 2012)^ |
| People who lived in the country and in a family, house appreciated the abundance of space in their residential milieu, because space guaranteed privacy (U) | "I have my own yard and freedom.”^pg. 252(Juvani et al., 2005)^ |
| The elderly felt that their homes and communities were free and comfortable places (U) | “In my house, I can do whatever I want. I go where I want to because no one stops me from doing that. But, you see, (in the facilities for the elderly) you have to eat what you are given, and when they tell you to sit still, you have to sit still. (informant 5).” ^pg. 101(Park & Ko, 2020)^ |
| Absence of community supports services influences an older adult's meaning of home and independence (U) | “The bank, the grocery, the pharmacy, [and] now we only have convenient stores. Before, 30 years ago, I could walk to the grocery, to the bank. But now that I’m aging and I would like to stay and to live in my apartment, I have to take my car to go to all these places: the grocery, the pharmacy, [and] the bank^.” pg.366(Bigonnesse et al., 2014)^ |
| Participants were reluctant to signal neediness and preferred to work through challenges rather than involve family (U) | “We're both quite independent, we'd hate to ask, my husband had cancer and he had to have treatment, and out friends just railed round, and I couldn't tell my family because they'd have flown to be with us and I didn't want that.” ^pg. 2550(Neville et al., 2021)^ |
| The incentive to move was linked to maintaining independence if she was no longer driving (U) | “There's a new retirement place going up, just started right in the middle of [name of town], I thought that would be the place to be, I could walk to the bridge club, I could walk to the library. If you were town, it would be brilliant.” ^pg. 2551(Neville et al., 2021)^ |
| Category 1.2- The right fit between an older person’s abilities and the environment | |
| Relocation occurred if they couldn't access safe transit options (C) | “Participants indicated they would have to move, thereby bringing significant disruption to their usual occupations. A disruption in mobility was seen as a change that would be too big to adapt to.”^pg. 153(Vrkljan et al., 2011)^ |
| There is a need for formal assistance to live independently and remain at home (U) | “As we age we lose some of our ability to do the necessary things, even little things (like changing a light bulb in a high ceiling). We worry . . .,” and “I am afraid to climb a ladder to clean the gutters on my roof.” ^pg. 232(Black et al., 2015)^ |
| Health status, changes in function and need for assistance was a reason for relocation (C) | “I will need to move if I should develop any other kind of disability.” ^pg. 385(Martin et al., 2019)^ |
| Strong attachments to the home made it difficult to be objective about decisions to leave the home as their physical health deteriorated and/or the functionality of the home decreased (U) | “Occupants were satisfied with their homes because they considered them to be a good match for their homes because they considered them to be a good match for their needs, and whilst aware that they were 'slowing down', were satisfied to remain in their homes for as long as they could be sustained within the home and do their homes for as long as they could be sustained^” pg.1698(Mackenzie et al., 2015)^ |
| Deciding to wait on home modifications created loss of independence (U) | “At my mother's home, the bathroom doesn't have any major or special modifications. I advised her to replace the bath with a shower, or at least to do something to it that would make it easier for her to get in and out. But it's something she hasn't done for the moment and I don't think that she will. It is not a problem of money, and when I suggest anything that might make things easier she says 'I'll be long dead before I will be able to manage with those sort of things, and what is the use of doing any major work to the house if tomorrow I will no longer be there?'^pg. 1291(Renaut et al., 2015)^ |
| Feeling unable to meet these demands and being reminded daily of things needing repair or worrying that things such as appliances would break down was described as frustrating (U) | “As a single woman, it's a bit difficult because you need...you see that needs fixing and that needs repairing.” ^pg. 260(Almevall et al., 2022)^ |
| Some of them wanted to keep a garden but wanted one small enough to manage (U) | “I like the thought of having a garden that I'm in control of, rather than it being in control of me.” ^pg. 1187(Burgess & Quinio, 2021)^ |
| Others explained they were not satisfied with the practicalities of retirement housing (U) | “They were typically out of town, or in very small towns, and not well connected to London.” ^pg. 1188(Burgess & Quinio, 2021)^ |
| Those who owned their home had maintenance obligations (U) | “Still in my house and that the maintenance has not become too much.” ^pg. 6(Dendle et al., 2021)7.1^ |

## Table 2: Synthesized finding 2- Finances and costs constrained decisions about housing

Abbreviation: (U) Unequivocal finding, (C) credible finding

| Finding | Illustration |
| --- | --- |
| Category 2.1- Financial resources were a factor when making housing decisions | |
| Health and finances factors determined ability to remain at home (U) | “Wondering where I will be able to live when my money and health require another place . . .,” and “The economy has affected all of us. It’s harder and harder to survive financially . . . didn’t expect us to live as long as we’re living . . .”^pg.232(Black et al., 2015)^ |
| Older people in Latvia and Hungary had to sign their home over to relatives due to financial and material burdens of home maintenance (U) | “I have never asked anyone for help, and I hope I never need to... I'm not in such a bad state yet that I'm in need of help. I can still manage alone, and my sister takes care of me. I don't have to depend on others ... My daughter will inherit the house. I've already had it put in good order, but if she didn't bother ot visit me and didn't help at all, I would give the house to whoever cared for me, perhaps to one of my grandchildren. But, as I say, I am not worried, I have a good family. Female participant, Hungary.”^pg. 7(Sixsmith et al., 2014)^ |
| Their home was not one of choice (U) | “Could say I don't like to caravan but that's all we ended up with you know and haven't got any money to go any further.” ^pg.1695(Mackenzie et al., 2015)^ |
| Housing is a determinant for health and a key component for quality of life (U) | “If I have to move from my home for some reasons, I don’t know where I would go. Everything is too expensive for my income. I cannot afford going elsewhere.” ^pg. 362(Bigonnesse et al., 2014)^ |
| Due to low levels of financial resources, not much can be done about the home environment to improve quality of life (U) | “'They say that I should move from here, but I'm not going to, I'm staying here. They don' realise that that I know my neighbours well here. Things could be made better here, but because they won't do them I shall have to live with it. I mean, they have put some small things in, like grab rails, but they don't want to install a shower.” ^pg. 1294(Renaut et al., 2015)^ |
| A couple does not see the point of asking the housing associated to modify the home as they expect it will be refused (U) | “The bath-tub, we installed that. The housing associated gave us permission, we had to pay for it ourselves. And now we don't even use it. We get washed in the sink, like our grandparents did! I suppose we could ask them to install a shower, but they don't always agree. They want to sell off their housing stick and this building is already up for sale. They are giving preference to the existing tenants, but how can we buy at our age?” ^pg. 1295(Renaut et al., 2015)^ |
| Many interviewees decided to buy a more expensive home to invest in the housing market, sometimes through the renovation of a house in the existing stock (U) | “It was meant initially as an investment, but after we'd been in the house for a few years, we realised we really liked it and the intention was to keep it, which was fortunate because the house market kind of collapsed.” ^pg. 1187(Burgess & Quinio, 2021)^ |
| Those who upsized for a cheaper price moved to a cheaper area (U) | “Property in Bristol at the time was half the price of property in London, so for half the price of our London flat we got a 3-bed penthouse overlooking the newly regenerated harbour area of Bristol.” ^pg.1188(Burgess & Quinio, 2021)^ |
| Concerns expressed for older people who had low incomes and may not have the same resources (U) | “If I had to go through the public system I would have got limited support. I've only been able to make changes because I've got the money behind me.” ^pg. 2549(Neville et al., 2021)^ |

## Table 3: Synthesized finding 3- Feelings of stigma regarding ageing and concern about being a burden

Abbreviation: (U) Unequivocal finding, (C) credible finding

| Findings | Illustrations |
| --- | --- |
| Category 3.1- Negative views associated with moving house | |
| Participants living away from family and friends struggled with loneliness and felt less rooted 'in place'. (C) | “What I am most afraid of is that I die inside my home, without anybody else living here or coming to the house, and that they won’t find me until I’ve been decomposing for a few days.” ^pg 775(Finlay et al., 2020)^ |
| No intention of moving homes or installing modifications despite architectural difficulties of accessing the home (U) | “It is difficult to know what is going to happen in life, at different stage in life, all these little things that happen and that are unexpected. We do talk about the steep steps as there has even been an accident by one of the neighbours who fell going down and didn't recover.”^pg. 1290(Renaut et al., 2015)^ |
| Home has an emotional attachment enabling older people wanting to stay with their familiar belongings (U) | “I've been here (in her apartment) for only 30 years! (laughs) Somebody told me if I moved to an old folk's home, I couldn't take anything with me, including my computer. To me, it would be like a prison. Even if I became totally blind, I hope someone could still come and help me here. I would like to die here, dammit. I don't want to go anywhere.” ^pg. 4(Narushima & Kawabata, 2020)^ |
| Leaving home means leaving the place where the family gathers and having to get rid of personal belonging (U) | “To have access to the same level of services, we would have to spend between $1,500 and $2,000 each month. It [the apartment] will be smaller, we would not be able to entertain the kids anymore, and I would not have my workshop to work downstairs. It would be a disaster.” ^pg. 370(Bigonnesse et al., 2014)^ |
| Older adults feeling forced to downsize due to their age (U) | “Personally, there’s a question I find important. Because we are old, it looks like they want to put us in such small space! But, why? Because we are old people? We want to breathe like everyone else!”^pg.364(Bigonnesse et al., 2014)^ |
| All wanted to stay in the community home of their choice as long as they could (U) | "Going into a home? That's be the end of me. And I mean it.”^pg. 1805(Grimmer et al., 2015)^ |
| Living in one's own home often drew contrasts with institutional facilities for older people (U) | “Well the first condition is to stay fit enough to be able to live on your own. And to live at home; I'd much rather live here at home than in some institution. (male, living alone, receiving daily home help).” ^pg. 54(Nosraty et al., 2015)^ |
| Concerns about poor support if relocation becomes necessary (U) | “Don't ever want nursing home. Quality of life would be miserable because I don't have big bucks. These places not really happy places if low income.” ^pg. 386(Martin et al., 2019)^ |
| The sense of freedom and independence gained from living at home would be lost with moving out of home. (U) | “I'm too attached to my home... I'm used to being independent and I don't think I could confine down to regulations and rules.". ^pg. 5(Hatcher et al., 2019)^ |
| Older people had concerns about isolation and loneliness (U) | “It’s difficult being alone so much. It’s depressing at times, but I manage. Weekends are the worst. That’s when I hate being alone.” ^pg. 6(Dupuis-Blanchard et al., 2015)^ |
| They did not want the building to become a nursing home (U) | “We don't want our guest room to be used for living-in caregivers, we don't want this to become a locked facility in any way that means people with Alzheimer or dementia will begin to wander ... we expect that they will be placed in a better facility than here. We are not going to turn into a nursing home.” ^pg. 414(Puplampu et al., 2020)^ |
| Reluctance to accept adaptations (U) | “You also get a lot of people who don’t want adaptations, and they will struggle on and...[. . .] It’s change. [. ..] [T]he change in the house and leaving the house to... To family.”^pg.5(Bailey et al., 2019)^ |
| Concerns with long-term facilities such as residential care facilities (U) | “You have to get up at a certain time, eat when they tell you, and go to bed early. I hope I die before going into a nursing home.” ^pg. 5(Dupuis-Blanchard et al., 2015)^ |
| Entering a nursing home is a fate worse than death (U) | “Really, I can't say I'll never go, but I hope I die rather than go into a nursing home or if I do go, I’ll be lost by then.” ^pg. 960(Gould et al., 2017)^ |
| Loneliness was described as a problematic aspect of home (U) | “The loneliness is difficult.... If I fall, I won't be able to get up.” ^pg. 258(Almevall et al., 2022)^ |
| A majority had not considered any properties marketed for people aged over 55, mostly because they perceived them as an option for much older people (U) | “We honestly didn't think about it. I'm 75, my wife is 73. We didn't even think we were old enough.” ^pg. 1187(Burgess & Quinio, 2021)^ |
| The decision to move was not taken lightly (U) | “'...you have to really start your life over again just when you have everything organised where you are.” ^pg. 4(Dendle et al., 2021)^ |
| For many, it was difficult to move around urban spaces, creating a sense of fear and anxiety in leaving the home, and compromising access to health and well-being supports for older people (U) | “...her life is taken up by getting to medical appointments for different things. So she'll have to go down the road towards Manchester to the hospital. She'll have to go, until very recently anyway, every Tues to the anticoagulation clinic down in Withington. And then go a different way to her podiatry appointment. And then somewhere else to her GP. Weeks can go passed when she's got an appointment like four days out of five maybe. And that basically takes up the whole day for her getting somewhere and getting back.” ^pg. 213(Woolrych et al., 2020)^ |
| Category 3.2- Individuals did not want to be a burden | |
| Older people opted against being a burden or avoided conflicts within laws (U) | “When he (only son) was in his 50s, his business failed. He thought about too much and had a stroke. He has four young children, so I moved out and I am now alone ... His wife is working now to support the whole family” ^pg. 529(Tan et al., 2015)^ |
| For elderly Koreans, living in one’s home by themselves seemed to be related to thoughts they did not want to burden their children (U) | “My son says that he will not send me to a nursing home (and will take care of me by himself), but I know the current era is not such a time, so I need to take control of my own life, I am not going to my son's house. Whether it is inevitable or not, once I go to my son's house, it will be heavy in my mind... I am satisfied (living alone in my house). I've got to live by myself, errrr” ^pg. 102(Park & Ko, 2020)^ |
| Participants want to delay having to depend on others because of the fear of becoming a burden on their families (U) | “There is an emotional problem, when the older people don’t feel attended . . . it is not about cooking the meals, doing the washing . . . As an example: “Daughter! take your mother to walk!” But, as she is always in a hurry, she cannot go. Here is where the emotional problems come . . . specially for the older people that live at home” ^(pg. 10)(Bosch-Farre et al., 2020)^ |
| Growing older with dignity and independence is important in ageing in place (U) | “Being self-sufficient and not becoming a burden on or dependent on others.” ^pg.232(Black et al., 2015)^ |
| Family desire to reciprocate care (C) | “I don't want to be a burden on my children, and I am willing to go to a nursing care facility, but my children say I should live with one of them.” ^pg. 386(Martin et al., 2019)^ |
| Several reflected on one day needing to move to specialized housing in order to avoid becoming too great a burden on their loved ones (U) | “It shouldn't be that I'm laying here and they [family members] have to come and...because they have their own life.... I'm grateful as long as I can keep living in my own. but w11hen they realize it's time, I will have to come to terms with that [moving].” ^pg. 257(Almevall et al., 2022)^ |
| Several had already relocated, which had resulted in such problems described as adverse to well-being (U) | “I have only lived here for a year, I haven't been able to figure out these things in the apartment.... It's been somewhat problematic getting to know things, the stove and...so it took some...it was difficult”. ^pg. 258(Almevall et al., 2022)^ |
| Category 3.3- Stigma | |
| Frequent adaptations to the home prevented falls (U) | “Falls are terrible... I take my cane; some people don’t because they are ashamed of it.” ^pg.16(Bosch-Farre et al., 2020)^ |
| Residential homes or supportive living were seen as signals of being ‘dependent’ of loosing quality of life (U) | “When you see these old people’s homes, and their whole... Chairs around a television set. Oh, God spare me that. Really, I think that’s... That must be the end, when you’re reduced to that kind of thing.” ^(pg.7(Bailey et al., 2019)^ |
| There were implications of designing for wheelchair accessibility (U) | “When the ramp was finished, this workman with a really loud voice called out “this is now a disability house!” really loudly—the whole street would have heard.” ^pg.206(Tanner et al., 2008)^ |
| Adaptations cause negative stereotypes of ageing and vulnerability (U) | “ You walk down the street, and the street says, “Vulnerable older person. Vulnerable older person.” A key safe or grab rails or the ramps or whatever they happen to be.” ^(pg.6)(Bailey et al., 2019)^ |
| Losing their home or relocating, is like losing control of their everyday lives (U) | “When you move in a senior home, you have rules to follow. You have more freedom when you live in your own home.” ^pg.373(Bigonnesse et al., 2014)^ |
| Participants thought using ambient assisted living could be demoralizing (U) | “Most older people have a lot of pride and independence and having the comprehensive sensor monitoring would be insulting, and intrusive ... the constant monitoring would be really depressing; it would remind me that I have a really serious problem."^. (pg. 108) (Mortenson et al., 2016)^ |
| Category 3.4- Acceptance of ageing impacted housing decisions | |
| Acceptance of life as it has become (U) | ‘‘I will accept being admitted to the nursing home when I need to go there – I hope.” ^(pg 285)(Bergland & Slettebø, 2018)^ |
| Past generations have been through many difficulties due to historical, thus would rather to adapt and endure future hardships (U) | “I try to be positive, tolerant and philosophical about things. I know the importance of keeping a good mentality.... I have limited economic resources; the government doesn't provide much convenience and I don't think I can rely on my children. I don't know. I'd better exercise more to keep healthy. In that I don't have to count on anyone. But you can never predict, can you? I just take one steps and look around before taking another. I dare not think too much about the future.”^pg. 197(Yu & Rosenberg, 2017)^ |
| Remaining in the same house to age in place is not necessarily a good thing. (U) | 'Well I think with (my husband), being in the one place it was a security for him. But he traded on it rather than looked beyond it as he deteriorated in his health.... And I think that would have been a disadvantage, because he got comfortable and he got so comfortable he didn't want to move. “^pg. 361(Wiles et al., 2012)^ |
| Older people learnt to accept events of life during ageing (U) | “Very much to accept the limitations, the limits . . . Even if you don’t have good health . . . Calmly.” ^pg. 6 (Bosch-Farre et al., 2020)^ |
| Dependency is a life condition that participants need to accept sooner (U) | “We are getting old, and we will need our children’s help, and I don’t think they can help us . . . hence, we will have to afford a place in an institution, and even though, it is very difficult to find a place” ^(pg.10)(Bosch-Farre et al., 2020)^ |
| Older adults accepted home adaptations as the reality of becoming old as it met their changing, mobility and health needs (U)) | “That was one of the reasons that I didn’t want it. Because... Because of the look of it. But then you’ve got to weigh up the benefits, and the benefits outweighed the... You know, you’ve got to forget about sort of the look of things and think what benefits it’s given you; you know. And now, I never notice... You never notice it.” ^(pg. 8)(Bailey et al., 2019)^ |
| The decisions to move was sometimes made in conjunction with family as needs, real or imagined, changed (U) | “'Just over a year ago because I was turning 90, all the family and I thought that if I didn't get my driver's licence I'd be better in [name of town] closer to everything.” ^pg. 2551(Neville et al., 2021)^ |

## Table 4: Synthesized finding 4- Positive and negative attitudes to future housing

Abbreviation: (U) Unequivocal finding, (C) credible finding

| Findings | Illustrations |
| --- | --- |
| Category 4.1: Individuals with positive attitudes to future housing | |
| Most participants indicated they were willing to trade personal privacy for the potential to remain at home (U) | “If (ambient assisted living) meant being able to stay... home longer, then privacy would go out the door.” ^pg. 109(Mortenson et al., 2016)^ |
| All participants had a strong desire to continue living at home (U) | "The vast majority ... of people would prefer to stay at home and (if Ambient assisted living is) an alternative to going to a nursing home, I'm sure 99 per cent of people would opt for that” ^pg. 107(Mortenson et al., 2016)^ |
| Reducing their belongings from large houses was a common challenge (U) | “To live where you don't have a lot of space to put stuff, and it was just harder. It's not a big deal now, but getting rid of my stuff and downsizing was really, really ...”^pg. 413(Puplampu et al., 2020)^ |
| Positive attitude towards retirement homes (U) | “I have already thought about it, if I can't manage to do things on my own anymore, then they should put me in a home. I don't have any children, and well, you have to be realistic, this would be the best solution.”^pg. 1296(Renaut et al., 2015)^ |
| Concern about isolation (U) | “I have no family within 600 miles. Although I hate to leave my friends and organisations here, I expect there will come a time when I will need to move south where I have family. “ ^pg. 386(Martin et al., 2019)^ |
| Aging in place is related to the capacity of older adults to gradually adapt their spaces to aging realities (U) | “My bedroom is on the second floor. Now, I am still in shape, but later [if I have health issues], I will have to install the bedroom on the main floor.”^pg.363(Bigonnesse et al., 2014)^ |
| Proactive steps were taken to adapt their actions and environments to achieve their goals (U) | "I mean I could do things and my strength's gone a lot, but I try to work my way around things and figure out how to do them differently."^pg. 1806(Grimmer et al., 2015)^ |
| Ambient assisted living systems could transform these sense of home (U) | “It would be ... like living in a nursing home in your own home.” ^pg. 109(Mortenson et al., 2016)^ |
| Older adults who qualify for home modifications couldn’t install the correct equipment installed (U) | “Often, one problem we have is that we are not able to modify an apartment. The person’s needs have changed, we want to keep her in her home, but we are not able to adapt the apartment. For some reasons, the equipment does not fit in the bath, the wheelchair does not circulate, and there is no security system, no alarm system. It [incapacity to modify an apartment] comes often with poverty. (Service provider).” ^pg. 363-364(Bigonnesse et al., 2014)^ |
| Relocating during middle age took place due to their experience of how their ageing parents had faced difficulties (U) | “'Sure we, wanted a bungalow, that's what we were looking for... because we said to ourselves, in the coming years ... my wife, she said to me, I can't see myself climbing up the stairs and doing the housework, it's too tiring'. And we have seen what has happened to my mother in law, who can't go upstairs to her bedroom anymore so she sleep in one of the downstairs rooms.” ^pg. 1293(Renaut et al., 2015)^ |
| Housing and urban environments adapted to older people was important (C) | “When we moved, the first thing I said: a shower.” ^pg. 16(Bosch-Farre et al., 2020)^ |
| Some people had their name on a hostel or aged accommodation reservation list as a kind of insurance for the future, but intending to move (U) | “I have my name down in 3 places... (so I can) keep my options open.”^pg. 1702(Mackenzie et al., 2015)^ |
| Spouses who were carers relocated to smaller homes and did not rule out the possibility of further moves, even to retirements homes (U) | “There is my bedroom and a separate bedroom for home where he has a hospital bed. Yes I have adapted the home, of course and especially the bathroom ... I won't move from here now unless I can get a housing associated flat on the ground floor and some more help for my husband. Otherwise we'll think about going to a retirement home where there will be more help and less problems.”^pg. 1992(Renaut et al., 2015)^ |
| Living in a nursing home, with children, getting a domestic helper as a caregiver were considered but not explored with their children (U) | “I was with a large group of friends when I told them that I would stay with my children. Some suggested a nursing home... I don't know, I do not understand what my children think...or maybe I should employ a domestic helper?”^pg. 530(Tan et al., 2015)^ |
| Word of mouth raises awareness of home adaptations amongst older people (U) | “And often older people might have experience of a friend or a neighbour who has received a piece of equipment. They might have been struggling, but might not have realised that there might be a solution to that. And then they sort of realise there might be something out there that can help me as well, so.” ^pg. 5(Bailey et al., 2019)^ |
| Participants felt they were able to exercise their freedom in the residence by having a choice on where to live, choosing when to move (U) | “Well, I am (in my 80s) now and we moved about 5 years ago. My wife and I were starting to experience our friends and relatives ending up being sick and having to move and we thought we should move. We like this idea of (seniors' cohousing) and we thought it was important that we choose where we moved.” ^pg. 412(Puplampu et al., 2020)^ |
| Some participants described the move as difficult at first, and then gradually becoming rooted, practically and emotionally, and thus making the new place a home, even if this sometimes took years (U) | “Initially, it was awful.... I moved from my house where I had so much to do...and when I moved here I had a lot of energy that I felt I had no outlet for here.... But that was five years ago and life changes ....Now I don't have as much energy and I'm more content living here.” ^pg. 260(Almevall et al., 2022)^ |
| Downsizing is considered in relation to the broader context of individuals' personal circumstances and changes associated with ageing (such as becoming grandparents) (U) | “My wife’s mother was quite poorly which meant we were travelling almost daily to see her, and we decided that was silly so we decided to move so we could be nearer. Also our three kids were there with our grandchildren, all living in the same town. So we thought it would be useful if we were there so we could enjoy grandparent duties.” ^pg. 1186(Burgess & Quinio, 2021)^ |
| For some, moving to a larger property was actually a way to anticipate future needs (U) | “We thought, 'well, we can manage', because of the size of the house, if necessary we could live downstairs”. ^pg. 1187(Burgess & Quinio, 2021)^ |
| Participants described valuing the freedom to relocate in the later seasons of their lives, recognising the need for their home to fit with their lifestyle and anticipating their needs were likely to change (U) | “'I would like to move out of the small town I live in and be closer to the city of Adelaide but not in it. All my children live in Adelaide and I currently live four hour away”. ^pg. 4(Dendle et al., 2021)^ |
| People living in single or couple households felt that maintenance demands could compel a relocation or downsize whether they wanted to or not (U) | “'Living in a very large house, just the two of us will be difficult in the future.” ^pg. 4(Dendle et al., 2021)^ |
| There were renters who valued the freedom to relocate or travel without being tied to a permanent home base (U) | “Probably rent for a while in a neighbouring town which is a bit more cosmopolitan.” ^pg. 5(Dendle et al., 2021)^ |
| Some participants had settled in early adulthood, perceiving it to be a good place to raise a family (U) | “We found that living on a wage in [name of city] with four boys was a quite a struggle ... we came up here to get away from the city.” ^pg. 2545(Neville et al., 2021)^ |
| Maintaining an older home was identified as more difficult for ageing bodies and this meant by having to reassess how future upkeep would be managed (U) | “The house is very old, it was built in the 1940s. Whilst we took on the job of maintaining it, it's getting a bit onerous because you know the roof now needs some new sheets. I'm getting a little bit old for doing that sort of stuff.” ^pg. 2551(Neville et al., 2021)^ |
| The decision to move into a flat in sheltered accommodation was made with her late husband (U) | “'We’d more or less rules out that we wouldn't move away from the area because we felt that friends were an essential part of life for us.” ^pg. 8(Webber et al., 2022)^ |
| A move from one house to another does not necessarily always disrupt a sense of home, rather living in sheltered accommodation offered the possibility of greater sociability (U) | “Having somebody to socially react with at home and to talk” ^pg. 8(Webber et al., 2022)^ |
| Category 4.2: Older adults who avoid thinking about future housing | |
| Don’t envisage making any modifications or adaptations despite health concerns (U) | “We'll wait and see what happens, is something happens to us, then we will have to make modifications, but for the moment, to get somebody to do something for something that might never happen, is it worth it? ... For my wife, at the moment she has some problems getting in and out of the bath, but for the moment there is no real problem, and if the time comes, there are always those rails that can be installed to help you get up.”^pg. 1290(Renaut et al., 2015)^ |
| Avoiding talking about future care needs to keep present circumstances (U) | “At my age, you know, as they say, I don’t buy any green bananas anymore, but I try to live day by day, not to try to plan too much in advance.” ^pg. 960(Gould et al., 2017)^ |
| No firm plans about changes in the future (U) | “Eventually we might have to get some assistance, I'm not fooling myself but at the moment I'm healthy enough to do most of the things I have do.”^pg .1701(Mackenzie et al., 2015)^ |
| Participants were unsure of how to start the environmental adaptation process or concerned with the high cost (U) | “Participant who received a ramp and a scooter, 'I didn't even realize, you know, that I wasn't doing it ... and now I can do it, you know, all this stuff and like just go to the store .. I had gone, all this stuff and like just go to the store ... I had gone, I go nowhere unless I have to ... and no I can scoot right over there and ... It makes a huge difference.” ^pg. 10(Shin et al., 2021)^ |
| Accepting limitations for emotional stability (U) | “And to not think much about the future, because you don’t know what will happen.”^pg. 6(Bosch-Farre et al., 2020)^ |
| The need to move in the future was considered when they could no longer take care of themselves (U) | “My mom is on the waiting list. Well, it's been 10 years already since she registered. It's one of the Chinese long-term care homes. Oh yes, it's common. They say it normally takes over 10 years!” ^pg. 4(Narushima & Kawabata, 2020)^ |
| Lack of awareness of how to access home adaptations (U) | “People actually don’t know that these services are out there. And also how to access them. You don’t get taught, at any point in your life, how to become an older person. It just sort of happens, [. . .] You know, if you have a child,... you’ve got your health visitor and they explain what you’re supposed to do. You become old and no-one is there telling you.” ^pg.5(Bailey et al., 2019)^ |
| No matter the circumstance, relocation will not occur and will not contemplate small modifications (U) | “I don't know... the time that it would take to install them.... and at the moment I can manage in the night on my own. All I really need is someone to give me a bit of help dressing, preparing meals and doing some cleaning, and I have already got someone to do that... so I think i'm ok.” ^pg. 1294(Renaut et al., 2015)^ |
| Those unconcerned about future plans were inattentive to changes or modifying their homes (U) | “I have never thought about them (home modifications). What would I need them for? No, for the moment and I don't need anything like that. Like everybody, I don't know what the future holds, what will happen, nobody can foretell the future, I might even fall on the stairs tomorrow coming back form the bakers.”^pg. 1290(Renaut et al., 2015)^ |
| No intention to move despite reduced mobility and declining health (C) | “Participant no 15 described it was like 'interia' after living in a place long enough. He would not think about moving, especially after he had a stroke and at least he knew how to go to hospital from where he lived.”^pg. 196(Yu & Rosenberg, 2017)^ |
| The demand of everyday life was a priority rather than thinking about future changes for them to age in place (U | 'Things don't bother me, I get washed in the sink, it takes me about an hour... there is a chair in the bathroom so I make do with that. In any case, my children have told me that it is not possible to install a shower, there is a window above the bath and it isn't possible to put a shower in front of a window.” ^pg. 1293-1294(Renaut et al., 2015)^ |
| Unaware of their current needs (U) | “A lot of the referrals come through from the care agencies. That are going in to see that person to provide care and the older person themselves has not been aware that there’s been something wrong or . . . They need”^pg. 5(Bailey et al., 2019)^ |
| Thinking day to day (U) | “I don’t think about the future, it’s day to day. When I get up in the morning, I put my two feet on the ground, and I thank God I’m able to put my two feet on the ground. It isn’t much, but that’s what it takes.”^pg. 960(Gould et al., 2017)^ |
| People had settled, or hoped to settle, into a home where they could live out their lives (U) | “'Really don't want to move... I had so many moves when we were young.” ^pg. 4(Dendle et al., 2021)^ |
| Observing the prospect of moving away could be scary for some (U) | “'very scary for some elderly people.” ^pg. 4(Dendle et al., 2021)^ |

## Table 5: Synthesized finding 5- Emotions, meaningful activities, and attachments with the home

Abbreviation: (U) Unequivocal finding, (C) credible finding

| Findings | Illustrations |
| --- | --- |
| Category 5.1: Emotions related to the home | |
| Taking care of their own home is a source of pride and joy for older people (U) | “I’m still doing it, rack[ing] the leaves and all that. I still have the capacity and I want to keep doing that kind of work to stay in shape. Because physical conditioning, I’m doing it but I’m a little less assiduous. So, when you have an obligation you force yourself to go get the newspapers and to work on your property. I’m really satisfied with my home; it’s the same since the past 40 years.” ^pg.372(Bigonnesse et al., 2014)^ |
| Knowing each other lead to trust, security and respect for each other (U) | “So, you know, I like that sense of community. You know, I would really give anybody my keys and say I am going on vacation, can you water the plants or whatever. And, I would give to one person but I would give it to anybody or give it to five people. You know, I really feel that sense of comfort and sense of trust.” ^pg. 411(Puplampu et al., 2020)^ |
| Comfort was one of the meanings of home (U) | “It's very comfortable...happy to be here and it is, with all my things around me, it's just like my own home." ^pg. 6(Hatcher et al., 2019)^ |
| Habits served to facilitate and maintain a strong relationship between occupations they enjoyed and their environment (U) | “I have to keep myself busy and I keep myself deliberately busy because it (volunteering) keeping me going ... it gets me out of bed in the morning^.” pg. 152(Vrkljan et al., 2011)^ |
| Living at home gave individuals a degree of purpose in life and a reason to keep busy (U) | "I feed my cat and look after that, keep the house tidy in general and I see that raking up's done out in the garden^." pg. 5(Hatcher et al., 2019)^ |
| Living at home and having access to their own garden provided comfortable place for relaxation (U) | "It's a sort of relaxation to go down there (garden)." ^pg. 6(Hatcher et al., 2019)^ |
| Home gave a sense of freedom and autonomy (U) | “I have freedom and I don’t have to answer to anybody . . . (I do) what I want to do in my garden, in my house, when I want to do, what I want to do and all that.”^pg. 43(de Jonge et al., 2011)^ |
| The meaning of home encompassed being free of constraints (U) | "Because I am sure that living in my own home, definitely (means) more freedom."^pg. 5(Hatcher et al., 2019)^ |
| Most participants valued their homes because of the sense of privacy they provided (U) | “I wouldn't want to be watched going in and out of the bathroom. That would be ... encroaching on (my) privacy.” ^pg. 109(Mortenson et al., 2016)^ |
| It was important for participants to in their homes as long as possible for familiarity. (U) | “No (aging in place doesn't have to mean being in the same place) but the likely advantage of staying in the same place, like me being in my own house for 29 years, that's a form of security. Because you're familiar with the background, you're familiar with the places. If anything goes wrong, and I have a private alarm. You feel that in your own home there's the contact that can come to you, and you know where things are.” ^pg. 361(Wiles et al., 2012)^ |
| Home was a place for revival and restoration after engaging with other in the community (U) | “Being at home between activities and social outings as you stay at home to catch your breath." ^pg. 6(Hatcher et al., 2019)^ |
| A comfortable home provided peace of mind and a sense of competence in a familiar environment (U) | "I know where things are so that's a big plus ... It's familiar". pg. 6 "I'm comfortable... I know where everything is that I need... even if I didn't have any fairly food eyesight I would still be able to get around." ^pg. 6(Hatcher et al., 2019)^ |
| By being able to manage at home gave older people a sense of existence (U) | “The most important with home is that I am independent, which means that I must force myself to manage. Even if it is hard I have to do it. And if I wouldn't manage it would be a mess, and I would not be able to live.” ^pg. 4(Sixsmith et al., 2014)^ |
| Home is a place of comfort and the importance of the environment surrounding the house to their experience of home (U) | “The garden, front garden and looking up at the sky and the back garden with the lovely birds . . . just sitting at the kitchen table and looking out at the garden at the birds.”^(pg. 43)(de Jonge et al., 2011)^ |
| Objects in the home provided a feeling of connectedness to the outside world (U) | “The things that are in the house have a meaning … this old chair was 20 pounds, the hall table there about 30 dollars, I had very little money and had to find gems among junk. So everything has a little story, really it does, a journey I went on to find things and how I made things work. And you feel filled up with the memories and the meaning of things, and content. I feel very blessed here. I think that when you’ve been independent you’re inclined to be thinking that you are alone. But somehow or other, in finding the meaning in things, I’ve come to the realisation that there’s a difference between isolation and solitude. And so I think that once you realise that you’re part of humankind.”^pg.44(Coleman & Wiles, 2020)^ |
| Home was referenced with a sense of identity through ownership (U) | "It is my home because I own it." ^pg. 4(Hatcher et al., 2019)^ |
| Comfort was one of the meanings of home (U) | “It's very comfortable...happy to be here and it is, with all my things around me, it's just like my own home." ^pg. 6(Hatcher et al., 2019)^ |
| Home was described as a place to feel free, comfort and enjoyment of your own privacy (U) | “Because at home I am free, comfortable, and I do prefer to be at home with my wife than in any institution.” ^pg.10(Bosch-Farre et al., 2020)^ |
| Being around people you trust was the reason to relocate (U) | “We knew we wanted to live in a situation somewhat similar to rural; the best of rural living is when you know all your neighbours.”^pg. 411(Puplampu et al., 2020)^ |
| Home was a source of emotional and physical comfort, peace of mind, stability and having a space for relaxation and restoration (U) | "It's very comfortable. We're here together... Out home is very important to us... I think we've got everything we want in this house." ^pg. 6(Hatcher et al., 2019)^ |
| Home offered a place where there is freedom to come and go (U) | "Come and go when you wish... without someone wanting you to do this or that." ^pg. 5(Hatcher et al., 2019)^ |
| The background and familiarity of the home provides security and an emotional attachment to the home (U) | “So I suppose the advantages of staying in the same place would be that you got to know people, that you were familiar with your surroundings, that your house probably had everything done to it, you wouldn't need to be doing all these things, you'd have it exactly as you wanted it for your lifestyle, so that would be an advantage. Whereas I seem to be forever shifting and making new gardens and painting houses and extending decks and redecorating so that nothing is quite as it should be... ” ^pg. 362(Wiles et al., 2012)^ |
| Home provided stability when the ownership of the house was secured (U | “It is good to have this house under my name. It feels good and comfortable to own a house.” ^pg.101(Park & Ko, 2020)^ |
| Home was a place of security (U) | “A safe feeling. You sort of go back to the old cave- the guy went into the cave, and it protected him from the weather and it protected him from the wildlife, it protected him from invasion.” ^pg. 107(Mortenson et al., 2016)^ |
| Their home had a strong sense of belonging (U) | "I feel more at home here than I would anywhere else I think. It take a long time to become acclimatised." ^pg.3(Lewis & Buffel, 2020)^ |
| Personalised objects were important characteristics of creating a home (U) | “That picture up there, (indicating a black and white photo of a man and woman), that was taken the day war was declared. See that one with the air force man? On the other side there’s a photo of a soldier and a girl? Well that’s G and I. I was 17, and he was 21. And that was the day war was declared. It was an open day at (E) camp Frasier’s paddock. At 5-o’clock in the afternoon, Churchill, it came over the loud speaker that had been declared....All the paintings you see around, our families have done those.”^pg.202(Tanner et al., 2008)^ |
| The home and objects in it also represented what had been accomplished during one's life, and this gave participants a sense of pride (U) | “It means a lot....all the things around me.... that I created from nothing...means that I was successful.” ^pg. 289(Almevall et al., 2022)^ |
| A minority spoke of their homes as places that made them feel constrained, or where they felt trapped, describing marital and co-resident relationships that had broken down, making their home into somewhere they longed to escape (U) | “'It does not feel as welcoming as it did when we moved in 20 years ago.” ^pg. 5(Dendle et al., 2021)^ |
| Neighbours who were noise, disrespectful, unhygienic, or violent could turn people's otherwise suitable home into a place they wished to leave as soon as possible (U) | “'Hoons (loud disrespectful drivers) doing burnouts in the street.” ^pg. 5(Dendle et al., 2021)^ |
| Some compelled to relocate could exacerbate an already distressing experience (U) | “'Drug-crazed ex neighbour.” ^pg. 5(Dendle et al., 2021)^ |
| Home was discussed as much more than physical building (U) | “Every day is busy and different and flexible and unexpected at times.” ^pg. 5(Dendle et al., 2021)^ |
| Great importance was made to ensure her house felt like a home (U) | “Like a home; homely and welcoming.” ^pg. 7(Webber et al., 2022)^ |
| Her life contributed to a deep sense of belonging to the local area (U) | “You do feel part of those communities.” ^pg. 8(Webber et al., 2022)^ |
| Isolation reveals the unmaking of home through the damage to the material and emotional components of home (U) | “I used to have [have friends round her house] but I don’t have any more.” ^pg. 10(Webber et al., 2022)^ |
| Home was seen as a place of emotional connection and psychological attachment (U) | “I’ve been here 43 years [I don't want to move]. That's how my daughter sees it when she comes back. That makes me feel good. It's the positive and negative over the years. Negative things are a part of life, how you overcome them. Other memories that have enlightened your life. That is home.” ^pg. 211(Woolrych et al., 2020)^ |
| Category 5.2- Meaningful activities within the home | |
| People who lived in their family houses often found gardening a therapeutic and pleasurable activity (U) | "My kitchen garden is the most important thing for me in the summer, the only therapy I need. I work in the garden whenever I feel up to it and watch the plants grow.”^pg. 251(Juvani et al., 2005)^ |
| Alternations accommodate changes in the family or older relatives living with them (C) | “Some changes over time were related to activities that people valued, such as having a decent workshop or electrical work to accommodate a computer installation”^(pg. 1699)(Mackenzie et al., 2015)^ |
| Having your own space in the home to accommodate a range of activities was important (U) | "See, we’ve got the front bedroom as our main bedroom, the second bedroom is Lara’s artist room, the third bedroom is my office . . . We’re using the whole house . . . It’s a seven-room house and we’re using them all.”^pg.43(de Jonge et al., 2011)^ |
| Living in a shared house did not provide a sense of self as you could not personalise the home (U) | “In an apartment, you’re not at home. You can’t paint and you can have a building manager that does not allow for any social activities.”^pg.5(Dupuis-Blanchard et al., 2015)^ |
| Freedom is negatively impacted when modifications weren’t installed for outdoor/garden access (U) | “I miss even being able to take my washing and hang it out myself .. . I can go out to the little balcony but it’s not the same as pottering outside and pulling a weed or cutting a flower or something, I miss that.” ^pg.127(Aplin et al., 2015)^ |
| Having one's own space where it was possible to close the door was described as a fundamental aspect of home (U) | “No, I don't like it when they try to stop me.... I guess they are concerned about me, but they don't need to be...so I do it anyway”. ^pg. 256(Almevall et al., 2022)^ |
| Category 5.3 -Strong sense of attachment with the home | |
| The investment of personal and financial resources in obtaining one’s home further strengthened the sense of attachment to their home (U) | "never ever thought about" living else where." ^pg. 4(Hatcher et al., 2019)^ |
| Would leave their homes 'in a box', or will remain at home (U) | “Would rather leave in a box or we'll stay here till the last day I'm sure of it”. ^pg. 1701(Mackenzie et al., 2015)^ |
| Older people voiced wanting to stay at home and others considered they were too old to consider moving (U) | “I just have a feeling that people will say that funny old lady that lives up there on the corner with those animal and that overgrown garden and they'll be all talking about me. Because I've made no arrangement to go anywhere or do anything.” ^pg. 1702(Mackenzie et al., 2015)^ |
| Older people were still living in their family homes, where they were raised (U) | “We had our twelve children here. It started as a nothing house – and it became our house . . . A home . . . We’ve decided what rooms we’d have and we built them over time.”^pg. 43-44(de Jonge et al., 2011)^ |
| Cherished possessions foster connection to the past while aging in place (U) | “And you feel filled up with the memories and the meaning of things, and content. I feel very blessed here. I think that when you’ve been independent you’re inclined to be thinking that you are alone. But somehow or other, in finding the meaning in things, I’ve come to the realisation that there’s a difference between isolation and solitude. And so I think that once you realise that you’re part of humankind.” ^pg.44(Brim et al., 2021)^ |
| Cherished possessions act as a mechanism for coping with physical and daily ageing changes (U) | “These (cherished possessions) show something that I was involved with and looking back on these I am absolutely delighted, I feel very grateful and very proud of myself. These days, sometimes I can’t do some things, there are a few things I can’t do, I’m not as steady on my feet as I used to be. Well, this is something I’ve done that makes me feel fantastic.” ^pg. 45(Coleman & Wiles, 2020)^ |
| Building the home and raising children in the home led to close attachment (U) | “Their attachment to the home related to having built the home themselves, for eg one couple 'just after the war, things were very hard to build and I think when it's your first home and you've really had to work for it and extend like we've done... you don't want to move because you've put so much of your life and you've brought your children up (in the home).” ^Pg. 1697(Mackenzie et al., 2015)^ |
| Objects of meaning help older people to connect to previous achievements (U) | “I’ve done a lot of amazing things in my boat and sometimes people don’t believe you’ve had the adventures you have had when they see you as just an old man. You say you caught a big fish, and they say “oh yeah” in disbelief. So, I can get out these records, the photographs and other things and show them. I also keep clippings in albums and up on the wall to remember things and to show to people.” ^pg.44(Coleman & Wiles, 2020)^ |
| Displayed cherished possessions in the home gave the sense of being surrounded by memories, people and the things they had done in their past list (U) | “For John, being surrounded by objects that speak about people and times I’ve had led to a strong feeling of being home, which can be interpreted as existential insideness.”^pg.43(Coleman & Wiles, 2020)^ |
| A part of them had gone into building and creating their home (U) | "We built our house." ^pg. 4(Grimmer et al., 2015)^ |
| Their home had a strong sense of belonging (U) | "I feel more at home here than I would anywhere else I think. It take a long time to become acclimatised." ^pg.3(Lewis & Buffel, 2020)^ |
| The longevity of domicile created strong and deep connections, resulting in a reluctance to leave (U) | "I couldn't imagine being anywhere else... I love my home and I lived there when I was married and I've never had another home on my own.. I don't want to leave it and I can't imagine going into a nursing home or a retirement village or anything like that because it's just, I suppose, just home to me^." pg. 4(Hatcher et al., 2019)^ |
| The home is a symbol of life and existence (C) | “One informant thought of her house as a place where others could remember her life, and she wished for her children to visit the house after she dies to remember her.” ^pg. 101(Park & Ko, 2020)^ |
| Home has a personal connection (U) | “The house she lives in now has been her home since she got married. This is important for her. She knows every little detail in her house and said: “And I am very fond of this place. I know every nook and cranny.”^pg.286-287(Bergland & Slettebø, 2018)^ |
| Home represented their past, present, future and gave the notion of anchoring self (U) | "But one's life gets locked into an area... your life gets locked up in the things you create."^pg. 4(Hatcher et al., 2019)^ |
| Cherished objects allow older people to connect with self and others (U) | “Let’s photograph that copper in the hallway with the old painting on it.. I found it in a bin outside an antique shop in Melbourne … I think it’s of the artist’s mother, but it reminds me of my own mother, she had copper hair. And not having had safe or loving family connections when I was growing up I found a happy connection to my mother through that plate, dear....Being drawn out from your own life is important, thinking about the past, difficult issues and bringing light to it, not being stuck in it, yes, finding some peace.”^pg. 45(Brim et al., 2021)^ |
| Dying at home was important (U) | “... when we are old and ready to return to our ancestors, it will be in this home. Not that we have old way of thinking. Young people can afford old people in there (nursing home) but it is miserable! You ask nine people, 10 want to return to their homes.”^pg. 530(Tan et al., 2015)^ |
| Cherished possessions brought back the past and what it presented to them ‘now’ to maintain continuity of self (C) | “For Rose, the very meaning of home entails nonhuman as well human others; she is “at home” because she is connected to things and people that reflect her life in both the past and present tense. Her connection to the past gives her a sense of continuity in the present.”^pg.44(Coleman & Wiles, 2020)^ |
| Personal skills and networks contributed practically to both sense of identity and ability to be autonomous (U) | “I’ve been in my house for 42 years. And had the same neighbours. . . . Yes. And so [the familiarity of the neighbourhood has] been marvellous for me. And the section is flat. And it’s been great! And that’s what I don’t want to lose [general agreement].” ^pg. 364(Wiles et al., 2012)^ |
| Having control with relocation by gradually moving (U) | “I am so attached to that farm. It is bred in my bones! I once thought that we would be able to make a transition slowly. I would slowly become involved in things, in this urban place, and to some extent it has happened, but I am resisting now. It is very interesting to be going back and forth. Tomorrow night I will be sitting in this house that I built with my own hands, in front of the fire and playing my music.”^pg.412(Puplampu et al., 2020)^ |
| People spoke of 'loving' their homes (U) | “Wouldn't go anywhere... we live in paradise already.” ^pg. 5(Dendle et al., 2021)^ |
| Felt a sense of belonging to the area (U) | “Very much so, especially this area, this road, it's very much a village feel... It's a long road and we all know one another^.” pg. 7(Webber et al., 2022)^ |
| Many older adults were bound to home and communities by shared memories, meanings and experience (positive and negative) within the context of place (U) | “In 1962, we were the first residents of this building, my father was the first one to enter that building, and then came the entrance where we are, the two of us came from the third floor and my neighbour is next door and we have almost like a fraternity, we were raised here so the neighbours all know us which we were little, that's a lot of family ... this neighbour of mine next door has been my neighbour for 54 years.” ^pg. 209(Woolrych et al., 2020)^ |

## Table 6: Synthesized finding 6- Safety, accessibility, and aesthetics in the home

Abbreviation: (U) Unequivocal finding, (C) credible finding

| Findings | Illustrations |
| --- | --- |
| Category 6.1-Individuals value safety and accessibility for their homes | |
| Ambient assisted living (AAL) would make them feel more secure in their homes. (U) | “Feel a lot more secure... because when you are experiencing various physical symptoms and live by yourself, it's easy to get a little but frightened of incidents happening and not be able to summon assistance.” ^pg. 107(Mortenson et al., 2016)^ |
| Being in a rental apartment did not make an informant feel safe after recovering from a fall (U) | “I feel more secured at home, I will not fall ... Just now, that woman (from the VWO) said that another resident had a fall; now she is in the hospital. (Previously) she told me to tell her in Cantonese, 'Do not walk about when there is no need. If you fall, there is no-one to help you.”^pg. 529(Tan et al., 2015)^ |
| Living in a home without access to an elevator became a reason for relocation (U) | “I’m seriously looking for a new place... it would not have stairs to clean. I find that is a waste of space and it’s a place where you can fall. We thought we would stay there for a long time but now my wife has problems with her hips. We were very satisfied with our home, but it has many levels and a lot of stairs. It’s not possible to reorganize it to only live on one floor. So, we seriously think about relocation.”^pg.363(Bigonnesse et al., 2014)^ |
| Modifications makes access and everyday life easier (U) | “I can actually not only invite visitors in now, I can actually invite clients in too and that’s another level again and that’s what I’ve been going for, is to be able to invite clients and that’s why I needed that separation, that’s why I needed it not to look like a hospital.” ^pg. 127(Aplin et al., 2015)^ |
| Modifications led to a safer environment (U) | It’s great, just great. They put grab rails all around. . . . [It’s] safer . . . [and] it’s more convenient too. It’s much more comfortable^. pg.204(Tanner et al., 2008)^ |
| The atmosphere and community around where the home was located was important (U) | “I have felt safe in this block of flats since I came here. There’s three single older women and a couple. There are five older women including myself and a young Asian couple and I just don’t feel threatened by the area.” ^pg. 43(de Jonge et al., 2011)^ |
| Older people, including family members attributed apartment living to feelings of security (U) | “We’d feel better if he was in a place where there were people around. At least if people haven’t seen him in a day or so, they’d check on him.” ^pg.5(Dupuis-Blanchard et al., 2015)^ |
| Older adults want a safe environment that includes a safety guard, night lightening and security systems (U) | “I wanted an apartment where I could feel safe, [a place where] if people want to come to my place they must buzz downstairs. Then I can answer directly and know that my door is locked. But it’s not everywhere you can find a building with this type of security. I will stay there for a long time!”^pg.365(Bigonnesse et al., 2014)^ |
| Home means being close to someone in case one needs help, safety, and security (U) | “I’m 85 now and have trouble with my sight and my back, walking in general, you soon get tired. I feel secure in that I can go in [to my neighbors] at any time for a little chat. And they are wonderfully kind. So I feel it’s secure round me too. I can go in and talk about how I feel*if anything has happened I can get it out of my system.’’ ^pg. 27(Dahlin-Ivanoff et al., 2007)^ |
| Modifications provided independence and freedom of choice (U) | “So basically to give Andy access in and out, we put an oven in so he can use it if he ever gets to that stage.” ^pg 105(Aplin et al., 2013)^ |
| Modifications for visitors, family and guests changed their engagement in social activities (U) | “I have a granddaughter who is in a wheelchair, and she came and stayed with me for a fortnight and she found she coped well, She said ‘Your bathroom’s terrific, Nana.”^pg 128(Aplin et al., 2015)^ |
| Modifications increased their independence to maintain habitual personal routines, and rely less on others (U) | “I’ve got more confidence and I don’t have to depend on somebody to be here in case I fall. It’s made me totally independent now^.” pg. 204(Tanner et al., 2008)^ |
| There was a need for a second grab bar because one was not enough (U) | “I have one shower bar on the right and to make the task easier, I need to get another bar on the left.” ^pg. 51(Owens et al., 2021)^ |
| Grab bars were used in video diaries to enter and exit bathtub (U) | “As long as I have the grab bar, and I have a good grip on the grab bar, I have no problems getting in and out of the tub.” pg. ^51(Owens et al., 2021)^ |
| Home adaptations supported mobility and health needs of older people to promote independence (U) | “100% get it done. It does change your life. Which, I’ll say, I’m a lot cleaner now to what I was. I’m a lot more independent in the house and everything else, so... Really, yes, 100%, get it done” ^pg. 8(Bailey et al., 2019)^ |
| All had some home adaptations by installing safety features (U) | “I’ve used it twice (emergency alert pendant or had installed an alert system with pull cord for their bathrooms) one time, they were able to get in through the kitchen window. The other time, I was doing Christmas decoration when my daughter phones, and when I turned, I fell. My daughter phoned a friend's husband to come, but before he arrived, I phoned the emergency alert and asked them if there was a particularly way he should pick me up. They immediately sent somebody and got me on the chair.” ^pg. 4(Narushima & Kawabata, 2020)^ |
| Adequate housing with ramps or bathroom adaptations allowed Inuit elders to deal with health conditions (U) | “Yeah, and I think the kitchen and the living room like this is ok, but having stairs for someone [with low mobility], like for old person, might be hard for stairs, [they should] be on a flat house.” ^pg.139(Baron et al., 2020)^ |
| Accessibility barriers such as stairs and lack of lifts were inhibited home access (U) | “I broke my leg, and I had no choice but to go up the stairs. You don’t know what I have been through with the stairs. I would raise a foot, and then the other I couldn’t raise it anymore, dragging, dragging, and raise the foot another bit again.” ^pg. 16(Bosch-Farre et al., 2020)^ |
| The need to downsize/home modifications (C) | “My home offers barriers: steps, narrow doors, remoteness to services.” pg. ^385(Martin et al., 2019)^ |
| Completing instrumental activities of daily living were deemed important to remain independent at home (U) | “Simple modifications to remain independent [made one] feel much more self-reliant.” ^pg. 232(Black et al., 2015)^ |
| Well planned housing is recognised (U) | “Well, for one thing I think this apartment is beautiful and well-planned. Then there’s an elevator and that’s good. I can’t go up and down stairs at the moment. There’s an elevator here and they’ve carried out alterations inside. Taken away the thresholds, and then there’s the world’s largest balcony I usually say, it stretches along the whole of the apartment and faces south/south-west.”^pg. 27(Dahlin-Ivanoff et al., 2007)^ |
| Home adaptations were crucial in enabling older people to live independently (U) | “We did an awful lot (on the home), we've been doing it since and ...now we got just lovely! I absolutely love it! I think it's just heaven! And erm I'd never want to go anywhere else, I just absolutely love it, I think it's ideal.” ^pg. 7(Sixsmith et al., 2014)^ |
| Older people value the convenience of the home and the layout of the home supported various occupations. (U) | “I mean having the carport on the same level I can just bring shopping straight in there. The upstairs laundry – it’s part of the house and I haven’t got to go downstairs to wash because with the washing machine that means you’re going up and down, up and down whereas here I can go on with something else and go back to the laundry again without any trouble.”^pg.42(de Jonge et al., 2011)^ |
| Preventative measured made to avoid social stigma with old age (U) | “Yes I arranged to have the second toilet put in upstairs, because we were not getting any younger and to have to go downstairs to the toilet at night ... so then we have to put the banisters in as well, because of going downstairs at the night ...”^pg. 1292(Renaut et al., 2015)^ |
| They were able to control their home environment as best they could (U) | “The very last thing I wanted to do was move into this building... Do I want to live here? No! But should I live here? Absolutely! ... If you think your health is going to be the same tomorrow as it is today, you are wrong. We all progress to some extent from day to day... I did not know the presence of a garbage disposal in the hallway was so convenient. So in the big picture, it was a very wise thing.” pg. ^4(Narushima & Kawabata, 2020)^ |
| Quality of life is good in the building because they did not have to worry about maintenance (U) | 'The quality of the building is good enough where you don't have to worry about doing maintenance; you don't have to worry about (the roof) falling down on you” ^(pg. 414)(Puplampu et al., 2020)^” |
| Choosing not to think about the future while still healthy (U) | “If I was in a wheelchair, I'd have trouble with the stairs.”^pg. 1702(Mackenzie et al., 2015)^ |
| Home modification decisions are important for future planning during ageing, deteriorating, or improving health (U) | “It’s good now that I’ve got the shower and its wide enough that if ever, I needed to put a wheelchair in there, its big enough for that.”^pg. 127(Aplin et al., 2015)^ |
| Older people explained they lived in a home which met their needs eg. Wheelchair accessible home (U) | “We lived in our own home. It was two levels with six steps to the lower level and 13 steps to the second floor. We just decided we can’t do this anymore. We sold the house and came to an apartment. pg. 4 One of the things I looked for in this home was the wide concept, to make it accessible if I ever had to be in a wheelchair.”^pg. 4(Dupuis-Blanchard et al., 2015)^ |
| There is interest in ageing in place despite age related changes (U) | “But each unit is contained on, you know, all the rooms; there is no stairs within a unit. So, that's the convenient factor if you are having mobility issues you can move around to every part of your own unit. And the elevator takes you from main floor to your floor and so, that element is important. And actually, the doorways are a little wider than normal. So, they could accommodate wheelchairs if necessary. The light switches are low, very low, lower than normal, so you could reach them from a wheelchair.” ^pg. 414(Puplampu et al., 2020)^ |
| Having a home they would not be compelled to leave enabled plans for modifications (U) | “'Modifications to bathrooms to make them more 'senior' friendly'.” ^pg. 4(Dendle et al., 2021)^ |
| A home which was a safe harbour was critically important for residents, also enabling them to welcome and nurture others (U) | “'A huge task... she has suffered a lot of trauma.” ^pg. 5(Dendle et al., 2021)^ |
| This feeling of security was a valued feature of the rural lifestyle they had chosen (U) | “'I feel really safe, I'd go out at night, think nothing of it.” ^pg. 2546(Neville et al., 2021)^ |
| Feeling safe was part of her lifestyle and was cherished (U) | “'I go to bed with the windows wide open, sometimes I get up and the front door is wide open, I love that feeling.” ^pg. 2546(Neville et al., 2021)^ |
| His flat was a space of safety that provided him with everything he needed (U) | “[do] what I want, when I want.” ^pg. 10(Webber et al., 2022)^ |
| The threat of physical barriers and impediments to getting around undermined older adults' sense of competence in terms of moving around the community (U) | “When one walks they stumble on the roads continuously since there are many rugged areas, gaps and holes in the roads. That is a great challenge indeed.” ^pg. 213(Woolrych et al., 2020)^ |
| Category 6.2- Older adults valued the aesthetics of their home | |
| Home is linked to identity (U) | “We actually think it (ramp) improves the look of the house.”^pg. 126(Aplin et al., 2015)^ |
| Home modifications can improve the look of the home (U) | “It looks really nice. Everybody remarks on it when they come in. They’ll say, Oh, it’s really nice. Uh-huh. The blue floor - it’s nice” ^(pg.8)(Bailey et al., 2019)^ |
| Changes to protect the home from the weather (C) | “Those stairs are pretty solid and the landing’s very good. But I had the awning put over it later. Because it sort of meant that the rain doesn’t come in any more.” ^(pg. 127(Aplin et al., 2015))^ |
| Home adaptations do not have a functional look (U) | “I really would have struggled to get in. Because there wasn’t a handle. And I don’t want a handle at the front door. Because I don’t like the look of it [a grab rail]. It’s like a pipe... A bit like a sewage pipe, you know what I mean?” ^(pg.8)(Bailey et al., 2019)^ |
| Home is a place where one can leave one’s mark and allow the place to look the way one wants it to look (U) | “A home is your own personal mark, so to speak ... I think carefully about where to put things and about colors. I have a feeling for both form and color. So to speak. It comes quite naturally to me. I always think of the balance in the living room. I think I’ve been quite successful with that. The main thing is knowing that it’s mine.”^pg. 29(Dahlin-Ivanoff et al., 2007)^ |
| Home is a reflection and expressions of self through decorating each new house with personal mementos (U) | "I can make a home anywhere... everybody has a few personal things that they dearly love.. I put up two paintings. That was home." ^pg. 5(Hatcher et al., 2019)^ |
| Appearance of the modifications (U) | “I guess to a degree we didn’t want it looking like a disabled bathroom, but it looks like a disabled bathroom I don’t know. So we paid extra for the tiles, a lot more extra tiles." ^(pg 105)(Aplin et al., 2013)^ |
| Lighting for elderly people was important as they felt that the lack of light influenced their psychic well-being (U) | “Then you just sleep (in bad weather). I feel that, in the dark period, I'm much slower and not willing to go anywhere, especially alone” ^pg. 250-251(Juvani et al., 2005)^ |
| Older people dislike the clinical appearance of grab rails as it remind them of hospital environments (U) | “With the best will in the world, adaptations can, you know, provide a very clinical... a more clinical environment, as assessed by need. And it’s trying to have that.... That, sort of, conversation with them about what is in their best interest, really. To keep them in the home, safe. And I think you have to be very sensitive to that. ^(pg.7)(Bailey et al., 2019)^ |
| Insulation, soundproofing, light and size of living was important (U) | “I have a nice apartment, I feel very comfortable... and there’s space. There’s light; it faces south. When I wake up in the morning, it’s sunny everywhere.” ^pg. 364(Bigonnesse et al., 2014)^ |
| Available space impact home modification decision making (U) | “It’s not only the expense, it’s just where you can put it (water lift) and how bulky it is and we don’t have a very wide block of ground so it’s hard to find a spot that would be suitable for it.”^pg 106(Aplin et al., 2013)^ |
| Home created into a personal meaning (U) | “We put extra cupboards up, varnished the floors, put curtains and blinds up ...just made it home.” p^g 202(Tanner et al., 2008)^ |
| Lack of control on the changes made to their home (U) | “They wanted to build something that looked like it was straight out of a hospital. It was shocking. It was horrible. It’s like they didn’t listen to a thing I said.” ^pg 105-106(Aplin et al., 2013)^ |

## Table 7: Synthesized finding 7- Family, community support whilst remaining at home

Abbreviation: (U) Unequivocal finding, (C) credible finding

| Findings | Illustrations |
| --- | --- |
| Category 7.1- Individuals felt the importance of access to essential community services | |
| Rural people found long distances an obstacle to their ability to live at home, because help was not easily available and they were tired of travelling long distances (U) | “Sometimes it is very difficult and I don't go (to the municipal centre) if I have a small health problem. I listen to my body quite long before I go to the doctor. Other people say that many would have gone much earlier. But one should not worry too much .” ^pg. 251(Juvani et al., 2005)^ |
| Accessing transportation was the link to the world beyond their home (C) | “Participants who did not drive relied on public transportation, including taxis and bus service, but felt these services required improvement to accommodate future needs.” ^pg. 153(Wiles et al., 2012)^ |
| Older adults found the home modification an uneasy process to understand due to multiple steps, causing them to hire their own contractor (U) | “If I need to install a grab bar in my bathroom, where should I go for that? You can ask your local health and social services clinic but it takes 3 months before [they contact you]... You have the time to fall!“ ^pg. 363(Bigonnesse et al., 2014)^ |
| Accessible transportation supported ageing in place (U) | “Everybody worries about it (transportation). My neighbor across the street is 77 and she might not be able to drive in a couple of years, and she says 'as soon as I can't drive, I've got to move.” ^pg. 153(Vrkljan et al., 2011)^ |
| There is an imbalance between formal and informal home care and support (U)) | “The agency working in this building has no personal support workers who speaks Chinese. For showering, communication is very important. That's why I need to translate. Otherwise I could be preparing breakfast during that time." ^pg. 5(Narushima & Kawabata, 2020)^ |
| Participant felt at home because she had good neighbours who often got in touch (U) | "I've been in the area so long, I'm quite well known. Even the kids say, 'Hello Mrs Smith, are you all right Mrs Smith, do you want me to carry your back?" pg. 3-4(Lewis & Buffel, 2020) |
| When the help is not available, relocation was considered (U) | “I think about moving because my home is too big. It’s a lot of maintenance. Then you ask yourself, because one time one of us has been sick you know? But you must mow the lawn! You have to maintain the house! What are you going to do? Then, you start to look for a new place. You’re forced to leave.” ^pg. 372(Bigonnesse et al., 2014)^ |
| Accessing transportation was the link to the world beyond their home (C) | “Participants who did not drive relied on public transportation, including taxis and bus service, but felt these services required improvement to accommodate future needs.” ^pg. 153(Wiles et al., 2012)^ |
| Living in a home that has access to public transportation was important (U) | “Well, the bus didn’t come within a mile to where she lived so she couldn’t get the groceries. There is a bus system, but it only goes here and there.” ^pg 7(Dupuis-Blanchard et al., 2015)^ |
| Living in a home that had access to other services such as health care services, meals and house cleaning was important (U) | “It’s nice here, but it’s far away from the doctors, the dentist, and other specialists.” ^pg. 5(Dupuis-Blanchard et al., 2015)^ |
| Being close to essential services was deemed important (U) | “They've got a small main street area with little grocery stores and the health food store, pharmacy, library... so if you're not carrying a lot of things, it's possible to get around town.” ^pg. 153(Vrkljan et al., 2011)^ |
| Most of our respondents were living alone, but it was important to have people around (U) | “Good age means you have people you know, that you do not have to be alone.” ^pg. 55(Nosraty et al., 2015)^ |
| Hard to make plans for old age (U) | “Everyday life consisted of little planning and little social life with others even though she has good friends. She continued to say that she did not make many plans but took decisions based on what she felt she needed at the time. Social life seems important, but it is limited to good friends living nearby, so it is possible to meet regularly.”^pg.285(Bergland & Slettebø, 2018)^ |
| There was great support in the building, including emotional, physical and financial support (U) | “We have actually been sort of taking care of each other. Just the first month we moved in, a woman on my floor who is still working was sick. She lives alone and she disappeared into her condo. And, 2 or 3 days after she disappeared in her condo, my husband and one of our retired nurses went to her door and banged on the door and got entry into her place and immediately took her to the hospital because she had really bad pneumonia. And, she was unaware of how sick she was. But again like it was the people in the community that said okay like we haven't seen her in 2 days, we got to check on her.” ^pg. 412(Puplampu et al., 2020)^ |
| Living with people of similar age group and political values brought happiness (U) | “I didn't expect our group to be so homogeneous, in terms of our political outlook. We are a non conservative group... it can feel quite isolating in out areas where we lived on a farm. Our farm (area) is very conservative.” ^pg. 411(Puplampu et al., 2020)^ |
| Opportunity for socialisation was a factor that contributed to improved mental health and quality of life. (U) | “There (are) so many opportunities to do things with other people here... go for lunch, go to a movie... I need the company of mostly other women and my husband really needs to be able to be around men. So today for instance he is just headed off to (another town) with our elderly friend Fred to help him move furniture into his cabin up there. He couldn't do without James' help, and James is just happy to do it. So just the impact on our life, we have friends here. We are not isolated in a big 5 bedroom house.” ^pg.412(Puplampu et al., 2020)^ |
| Accessible transportation supported ageing in place (U) | “Everybody worries about it (transportation). My neighbor across the street is 77 and she might not be able to drive in a couple of years, and she says 'as soon as I can't drive, I've got to move.” ^pg. 153(Vrkljan et al., 2011)^ |
| A home located in an area with access to good internet, mobile phone, or equivalent technologies for staying connected was vital for engaging in valued activities (U) | “'[free access to city-wide wifi enabled her to be] connected to information as I move.” ^pg. 6(Dendle et al., 2021)^ |
| Online access was particularly valued by people found it difficult or impossible to leave the dwelling due to health or logistic issues (U) | “'[I don't] get out very often and [I] love to catch up with what is happening around the world.” ^pg. 6(Dendle et al., 2021)^ |
| Home in location with inadequate public transport and walkability constrained what meaningful activities people were able to do (U) | “When I can no longer drive a problem arises it's too far to walk.” ^pg. 7(Dendle et al., 2021)^ |
| Even where public transport is available a mismatch between availability and desired activities is very limiting (U) | “The buses mostly run hourly, and stop at 9pm or earlier, because everyone has a car in this suburb.” ^pg. 7(Dendle et al., 2021)^ |
| Participants indicated that loneliness could be prevented by keeping busy and being socially engaged in the community (U | “You can find your own level quite happily, it's all about choices I've made, so that's the thing you do what you can.”. ^pg. 2549(Neville et al., 2021)^ |
| Relying on hired help for housework, gardening and odd jobs were ways some participants coped with more challenging tasks (U) | “I have a lady that comes in a does the housework, I have a gentleman who does the lawns and a farm manager who keeps an eye on the stock.” ^pg. 2550(Neville et al., 2021)^ |
| Some relied on their ability to walk to the local shops and services as they were no longer driving (U) | “It's not easy for me to walk down the street these days, it used to be but it isn't these days but I do it. I often have to give a leg a bit of rest in the afternoon for a little bit but that's okay, I do that. And I go in the morning when I've got a little but more energy.” ^pg. 2550(Neville et al., 2021)^ |
| A lack of seamless transportation options for older adults resulted in older adults having to take longer and more arduous journeys to get to their destinations (U) | “We do not have a metro station in Behala. if we need to travel by metro then we have to go to Tollygune or Kalighat metro station which takes more than half an hour, this is quite challenging for us... the journey becomes quite hectic at least for older people like us.” ^pg. 214(Woolrych et al., 2020)^ |
| Category 7.2-Older adults felt the importance of being close or connected to family | |
| Family members were the primary source for a wide range of household chores (U**)** | “It helps me a lot that my son and daughter-in law live here (in the same city). I've been calling them to do things. He installed the railing on the basement stairs, because I've had 3 falls since last December. It just makes me feel more secure. And my daughter-in-law takes me to a rheumatologist in another city, because I don't drive highways anymore.” pg. 5(Narushima & Kawabata, 2020) |
| Living nearby formal and informal help by family members and neighbours was important (U | “I have a friend who lives next door, she’s 39 and she does a lot of nice things for me. She’s a hairstylist, but she’s also a bank manager. She cuts my hair and she won’t let me pay her. She treats me like a parent.” ^pg. 5(Dupuis-Blanchard et al., 2015)^ |
| Family decided to live in her mother's home and put into place a strategy to ensure she ages in place (U) | “No, I live only for the present. you know, it is really difficult to think about what life would be like at my mother's age ... but I see myself staying in this house. You know, I have converted a couple of the rooms downstairs into a studio flat, with a bathroom. When my mother is no longer here I can rent it out and then after^.” pg. 1296(Renaut et al., 2015)^ |
| Older people valued their spare rooms as necessary for allowing children and grandchildren to visit (U) | “It's a home for my family... we've got 12 grandchildren, they know they they're always welcome to come and stay.” ^pg.1695(Mackenzie et al., 2015)^ |
| Being around family and neighbours were important (U) | “I’ve got all my network of friends in the area and I wouldn’t like to have to move right away at this stage and sort of make new friends again. I’ve got all my support group around this area. So that’s why I stay really.” ^pg.43(de Jonge et al., 2011)^ |
| Home provided a support network and prevented isolation allowing them to keep in contact with others (U) | "We have got our family around there. We got our interests all round this area.. It's just in a good position." ^pg.6(Hatcher et al., 2019)^ |
| Home was a place where the children came to see them (U) | “My youngest kid likes the side dishes that I've made for him. So, when he (my youngest son) comes to my house, I prepare some greens and side dishes for him to take home.” ^pg. 102(Park & Ko, 2020)^ |
| Decisions were made to move their family members into a retirement home (U) | “When she started to lose her sight, I talked things over with my brother and the only solution was that I should go and live with her. There was no question of her going into a home on that we were clear.” ^pg. 1296(Renaut et al., 2015)^ |
| Outdoor areas allow older people to retain meaningful occupations such as gardening (U) | “We have a proper house here with three bedrooms, which mean we can have our grandchildren back here to stay. So the house itself is ideal.”^pg. 43(de Jonge et al., 2011)^ |
| Widows relied on their families and neighbours to provide regular check ins (U) | “When she (neighbour) look up (from her apartment) she can see me. When she notices I do not open my window she will telephone me. I advised my son (adopted son in law), 'you call me on the phone in the morning and in the evening. If something happens to me at night, you will know in the morning. This way it is fine. If something happens to me in the day, when you call at night, you will also know' ... I fell very at ease.” ^pg. 530(Tan et al., 2015)^ |
| Seniors expressed concerns about not being able to provide food and lodging for family members. (U) | “I like to have food when the children come because they often have a meal here and on weekend's there's always visitors.” ^pg. 961(Gould et al., 2017)^ |
| Remaining in their homes was contingent on having strong social connections (U) | “I just look forward to Masha and Helen visiting me, there’s something really nice about having my family…they are all sort of close, they keep me company and in touch with what’s going on.” ^pg. 263(Neville et al., 2016)^ |
| Home is the place where social roles are continued and a place for family to gather (U) | “The home where I live is very comfortable; I really take care of it. It’s very functional; I feel content. I have space to entertain my kids and grandkids.” ^pg.370(Bigonnesse et al., 2014)^ |
| Family members lived far away, friends and neighbors were crucial sources of social support (U) | “When I had the cancer, I had radiation 28 times in December. Every morning I told my friends, I cannot do it one more day, but I did thanks to them." ^pg. 5(Narushima & Kawabata, 2020)^ |
| Home was a place to allow family to take over the ancestral rites (U | “Even after I die, I hope my children will not sell this house and just come and go like their hometown... This house has let me live my life. That's the meaning of this house.” ^pg. 101(Park & Ko, 2020)^ |
| Older people wish to remain at home as long as possible provided they were given access to home care services (U) | “If my health should require help with that, I would like the public health care system to give me such services. That I can get out and maintain contact with others.” ^pg. 4(Fjell et al., 2021)^ |
| Home provided a place, a time and a reason for positive contact with others as well as being where friends and family were close, which was described as central to well-being (U) | “It's simply that I have a home that provides me a my dog and my friends with a space that we share.” ^pg. 258(Almevall et al., 2022)^ |
| People described weekly dinners held at their home over many year brought together their children and grandchildren, a routine that they consciously continued to keep family ties strong (U) | “'I have four different lunch groups. We go to different places each time.” ^pg. 6(Dendle et al., 2021)^ |
| Having supportive friends and family nearby meant that they felt free to travel and visit relatives and friends without worrying about their home while they were away (U) | “Our neighbours keep an eye on it, put out the garbage, water the pot plants and check the mail if we are going on an extended holiday. We do the same for them.” ^pg. 6(Dendle et al., 2021)^ |
| Having family nearby was integral to their social and support network (U) | “It's nice to have a little bit of family close by because, although you have a lot of friends, inevitable I believe it's your family that are there for you at the end.” ^pg. 2547(Neville et al., 2021)^ |
| With reduced mobility, there was great comfort from having his family nearby (U) | “We have a daughter jsut down the road and another in [name of city] so it makes life very pleasant and of course family nearby is quite massive. We know people around here and all the families are all overseas, they're all on their own. I can see that getting progressively worse which means there will be more need for help.” ^pg. 2548(Neville et al., 2021)^ |
| Dwindling visits from his children and friends, contributed to his home becoming less porous and more isolating (U) | “It's why I'm going to be depressed when you go, I don't really do anything.” ^pg. 11(Webber et al., 2022)^ |
| Category 7.3- Older adults felt a sense of attachment towards their community | |
| Occupants described the importance they placed on their home's close location to community activities, family members, friends and neighbours (U) | “There's a good community spirit here' and support mechanisms within their neighbourhood 'we've got (name) over the fence, the neighbour, he looks after the place if we should go away and likewise we look after his place.” ^pg.1697(Mackenzie et al., 2015)^ |
| Older people were happy with their current home as it provided connection with community services (U) | “Well the house is central in position; it’s very close to the shops and transportation which is very good. We have got the railway of course; there is a good bus service along here.” ^pg. 43(de Jonge et al., 2011)^ |
| Location of housing was important in being able to access neighbourhood facilities with easy access (U) | “I would hold (the house) and would put it here, in the centre of the village, because I have to go uphill and it is getting more difficult every day.” ^pg. 17(Bosch-Farre et al., 2020)^ |
| Being with familiar people and the neighbourhood, supported their living arrangements (U) | “He (the son) asked me over. I said no. When my son goes to school, there is no-one at home ... The neighbours lock their doors. It is boring to be alone. Sometimes I went to the void desk (the vacant space on the ground floor of an apartment block that is often reserved for communal activities) and there is no one to talk to. It is also a long walk just to take a bus ... Here, there are many people and also food is readily available. Convenient! At Sengkang (a new surburban), one needs to travel a long distance just to get food. Sometimes, when I stay here, I have to cook for myself.” ^pg. 529(Tan et al., 2015)^ |
| Living in apartments or condominiums expressed contentment with their new home (U) | “I want to stay in my condo as long as possible because I'm comfortable, I'm close to everything and I have good neighbours. ^pg. 961(Gould et al., 2017)^ |
| Having access to health services was important for people who suffered chronic illness (U) | “About the community events, feasts, and so on, are there enough activities like that in the community? [Through an interpreter]: Yeah. Why is it important, why is it good? [Through an interpreter]: He says it’s important going, because it’s so boring here and sometimes [there is] hardly any food. So, it’s his only chance to go eat around people and socialize with others.” ^pg.140(Hatcher et al., 2019)^ |
| Location of housing was important in being able to access neighbourhood facilities with easy access (U) | “I would hold (the house) and would put it here, in the centre of the village, because I have to go uphill and it is getting more difficult every day.” ^pg. 17(Bosch-Farre et al., 2020)^ |
| Participants found reasons 'to get up in the morning' through routines around their neighbourhoods and broader communities. (U) | “‘It helps me keep going. I think now if I didn’t do this, I would probably be crippled. Just sitting around doing nothing. I have to get out.” ^pg. 777(Finlay et al., 2020)^ |
| Neighbours were referred as family (U) | “Researchers (people in my neighbourhood) are like a family. They call 119 (911) if I become sick. When I am sick, they come and see if I am OK, and if I'm not home, they look for me and ask around.”^pg. 101(Park & Ko, 2020)^ |
| Local social networks enabled participants to feel rooted in the community (U) | “I have very good friends. I’ve been widowed since 1978, and had I not had those friends, it would have been very difficult for me. And then they’re like family … very close good friends that care about you.” ^pg. 774(Fjell et al., 2021)^ |
| Older adults felt the need to consider a neighbourhood adapted to their needs as a preventative strategy (U) | “[My wife and I] have already established relocation criteria. We want to have access to community-based services close to home; [this alone is proving to be an] “endangered species.” We want public transportation to be accessible and efficient. It [the new apartment] must be a one-floor unit and it has to be close to green spaces. We want to go out for walks.” ^pg.365(Bigonnesse et al., 2014)^ |
| Older people stayed in their neighborhoods to avoid dealing with life struggles or interacting with their social surroundings. (U) | “I barely get out of my room now. For one thing my health condition makes it a bit difficult for me to move around. But I just avoid interacting with anyone beside my partner, unless I have to. I simply don't want to talk to anyone in the neighbourhood. I stay at home every day, do some reading and writing. I lose my temper sometimes with my partner. I know it's no good.”^pg. 197(Yu & Rosenberg, 2017)^ |
| Living in public housing remade neighborhood space into a more welcoming place that gave them a sense of continuity. (U) | “Since Danwei is now part of society, we belong to the community. No one is responsible for us anymore.... Some of us old neighbours still care about each other and the Community. Look at these chairs, tables and games! We bought them. We made those cushions as well. .... We volunteer in the neighbourhood and keep each other informed about recent developments. If we don't see the old folks showing up within a day or two we will knock on the door. We pay special attention to the frail ones who live alone.”^pg. 196(Yu & Rosenberg, 2017)^ |
| Quality of life had either improved or been sustained because of the motivations and activity in the community (U) | “I have improved physically because I am doing a whole bunch of new stuff with a bunch of new people here. I think for me a lot of it is what I mentioned before about the inspiration from other people here that have got me thinking about how do I want to go into old age. And I look at them and I think okay, most of them are in good shape, both physically and mentally. Some people aren't in great shape physically, but they still do stuff as much as they can. I think, that for me, that's the most important thing that has helped me.”^pg.412(Puplampu et al., 2020)^ |
| Having a community near the home was a source of support for older people that promote independence (U) | “She loves to go to the bank because three or four of the staff members, as soon as she walks in, they say hello. She has this personal connection with them; that’s a really big thing for her.” Older participants commented on the importance of having services available in their community: “We’re in a very fortunate area. The bank, the liquor store, the grocery store; they’re all right there. The pharmacy is across the street.”^pg. 6(Dupuis-Blanchard et al., 2015)^ |
| Home is a place for connection with their neighbours (U) | “Everybody helps each other; when one is staking wood, another one comes to give a hand. When I shovel my driveway, somebody will come to help me. When my neighbour sits on her porch—she’s old, she’s 90 years old—I go [to] her place and talk with her, she’s happy. My street is really like a big family.”^pg. 368(Bigonnesse et al., 2014)^ |
| Habitual occupations provided opportunities to connect with others (U) | “I go to town because I like the walk, and I meet people, and I like to talk to people, so I socialize often.” ^pg. 153(Vrkljan et al., 2011)^ |
| Elderly people in the community spending much time with people they were close to (U) | “I like it when my friends come to my house. When they come to my house, researchers drink tea and eat something, and researchers visit with one another. (When they come) I give them tea and fruit or something like that. I also go their homes, and they do the same for me.” ^pg. 102(Park & Ko, 2020)^ |
| Being close to essential services was deemed important (U) | “They've got a small main street area with little grocery stores and the health food store, pharmacy, library... so if you're not carrying a lot of things, it's possible to get around town.” ^pg. 153(Vrkljan et al., 2011)^ |
| Living at home enabled the older persons to maintain this sense of identity within their community (U) | "Those people (neighbours) had known him (her husband) and that is very important to me, that I am not just me but that I was part of a double, part of something else." ^pg. 4(Hatcher et al., 2019)^ |
| Easy access to grocery stores, medical sites, etc, were important to live near (U) | “If I get really depressed or down, I leave the building. Get in my car and go. I go to a lot of thrift shops, [food shelves], Goodwill, or just browse around [the local department store]. Brenda felt ‘at home’ in these shops where she was greeted by familiar employees and storefronts.” ^pg.772(Finlay et al., 2020)^ |
| Social networks contributed to the essence of what home meant and provided participants a sense of identity (U) | “I enjoy it here. The fact . . . [that] I have about 8 or 10 kids come of a morning now that school’s started. They wait here; if it’s raining, they wait on the veranda and otherwise they’ll wait in the yard and go out when the bus comes for the children ...and I like to be able to make sure they’re on the bus safe. Some of them get off here in the afternoon and the rest will get off in front of their place or another stop further down. But they all like to get on here. . . . By them being here the bus comes along up the road here, they walk across to catch it and I know they’re safe. ...It makes you feel you’re doing something even though I’m not really doing anything ...to most of the neighborhood children I’m “Nanna.” It doesn’t matter whether they are related or not. I’m Nanna. Even the 18- and 19-year-olds ...still refer to me as Nanna. I’ve got a very large family!”^pg.203(Tanner et al., 2008)^ |
| Social interaction for those who lived alone near younger people was valued (U) | “I think it's essential when you're designing something for us old people that are in close proximity to the young ... don't isolate us.” ^pg. 156(Vrkljan et al., 2011)^ |
| Proximity and depth of social connections were major factors in any sense of rootedness (C) | “I’m comfortable. I feel safe here. I was born here – I’m probably only three or four miles away from where I grew up. I know lots of people. My church is here; my whole life is here.” ^pg. 776(Finlay et al., 2020)^ |
| Older people coped with relocation and isolation by using public transit to go to the old city or their neighbourhood every day for familiarity (U) | “Participant no. 24 described how in her new neighborhood, no one talks to each other. She took three hour bus rides everyday just to visit the park in the old neighborhood.” ^Pg.197 (Yu & Rosenberg, 2017)^ |
| Individuals stated that they would prefer to continue living in an area with younger people rather than only with seniors (U) | “I didn't want to go into that sort of environment. I wanted to have younger people around me, I find that more lively. I don't want to go into care home type of atmosphere.” ^pg 1188(Burgess & Quinio, 2021)^ |
| The value of familiar neighbours and surroundings by expressing a wish that a quality nursing home could be built in her area (U) | “[I] could live out my days in the neighbourhood I've lived in for over 40 years”. ^pg. 4(Dendle et al., 2021)^ |
| Value was placed on the town's 'village' atmosphere and the friendliness of people (U) | “Small places are easlier to get to know people.” ^pg. 2546(Neville et al., 2021)^ |
| The care and friendship associated with being part of a small-town community was especially evident during critical and difficult times (U) | “'After my husband died there were people sort of turning up doing things, it was absolutely amazing.” ^pg. 2546(Neville et al., 2021)^ |
| Friendships were valued as they formed shared experiences of living on a remote peninsula (U) | “'It's a peninsula, there's only one road in and one road out, and because of that you're isolated in many ways, like our telephone, we can't have mobile reception because of the geology of the last, so you have to check on your neighbours, you can't ring them up, and if there's a civil defence emergency, which we're involved in, everyone actually has to go and make sure that people are okay, so you immediately have a warmer, closer relationship with people, but I think that's unique because we've worked on that really hard, that we've developed things that we do, which the whole community is involved in.” ^pg.2547(Neville et al., 2021)^ |
| Fostering good relationships with neighbours was considered instrumental in maintaining a supportive community (U) | “'I have got good neighbours, very nice, we all look out for each other but we are not in each other's pockets all the time.” ^pg. 2547(Neville et al., 2021)^ |
| Fitting in to the community was a two-way process (U) | “'We let our neighbour graze and in return he helps my husband with hedges. He put in the electric fences.” ^pg. 2547(Neville et al., 2021)^ |
| It took time and effort to feel accepting as a new comer in the community (U) | “This is a cliquey community, hard to break into anything. I did yoga for a year and honestly I was an outsider for the whole year.” ^pg. 2547(Neville et al., 2021)^ |
| Participants typically chose to engage in their communities in ways that matched their cultural preferences and enhanced their sense of belonging (U) | “We go down to the bowling club quite often to have a meal at night time and that's where all the people in my generation are.” ^pg. 2548(Neville et al., 2021)^ |
| Continuing to feel part of the wider community balanced what was perceived to be a limitation of an age-segregated community (U) | “Plenty here to do if you want to but I’m still trying to do things out of the community as well because I think that keeps you going and well that's good for your health.” ^pg. 2551(Neville et al., 2021)^ |
| Having lived in the same neighbourhood for most of her life cultivated long-standing connections with other residents (U) | “Being out, seeing people, talking to them.” ^pg. 7(Webber et al., 2022)^ |
| Her sense of belonging to her home was reinforced not only by her sense of belonging to the wider neighbourhood, but also strongly connected to her sense of community within the sheltered accommodation (U) | “There's a certain of social activity within the building which you wouldn't get in a long house, which is good.” ^pg. 8(Webber et al., 2022)^ |
| Just being present in the neighbourhood over a number of years and 'coping' with the changes was enough to form a sense of emotional attachment (U) | “I am emotionally attached to this community. Though I lived here for many years, I have not personally contributed anything here... We have coped with the changes in the community.” ^pg.210(Woolrych et al., 2020)^ |
| A strong-aspect of those experiences in the lower-income communities of India and Brazil was a sense of connection to home and community despite the everyday issues people experienced (U) | “I was born and brought up here. Where people are born they have strong connections and feel attached to their place. Even if neighbours are not pleasant we still feel belongingness as they are part of our neighbourhood.” ^pg. 210(Woolrych et al., 2020)^ |
| A number of older adults, even when they had spent a considerable amount of time outside of the area, reported a strong desire to return to their community in old age (U) | “I was always in love with Pelotas, I lived in Porto Alegre, I did not like it. I lived in Parana, I do not like it. I came back here. My place is Pelotas.” ^pg. 210(Woolrych et al., 2020)^ |
| Older adults reflected on the norms, values and attitudes which had traditionally orientated people to the community (U) | “Well everybody knew everybody else. Not anymore. Not the same. No sense of community. Definitely not. It starts to change the way you see the place, yet know, it’s not the same sense of connection that we all have. You used to know where you were. You felt centred if you know that I mean. I'm not sure communities know where they are from anymore. ^pg. 211(Woolrych et al., 2020)^ |
| Home was a place that people 'wanted to return to', an environment where social connections were sustained and where personal freedoms could be enjoyed (U) | “I was born and brought up here and feel attached with the people and this place. I can go out for some time but at the end of the day I want to come back here only. At this stage it is difficult to go to some new place and making new friends would not be easy.” ^pg. 212(Woolrych et al., 2020)^ |
| The feeling of 'being known' and recognised at a street level created a sense of belongingness (U) | “I know each and everyone here. They're very close to me. So, I definitely bear a different kind of emotional attachment with the locality and people of the locality. Everybody is known. Every face is known.” ^pg. 217(Woolrych et al., 2020)^ |
